# JAK-STAT activation contributes to cytotoxic T cell–mediated basal cell death in human chronic lung allograft dysfunction

**DOI:** 10.1172/jci.insight.167082

**Published:** 2023-03-22

**Authors:** Aaditya Khatri, Jamie L. Todd, Fran L. Kelly, Andrew Nagler, Zhicheng Ji, Vaibhav Jain, Simon G. Gregory, Kent J. Weinhold, Scott M. Palmer

**Affiliations:** 1 Department of Medicine, Duke University Medical Center, Durham, North Carolina, USA.; 2 Department of Medicine, Division of Pulmonary, Allergy and Critical Care Medicine, Duke University Medical Center, Durham, North Carolina, USA.; 3 Duke Clinical Research Institute, Duke University, Durham, North Carolina, USA.; 4 Department of Biostatistics and Bioinformatics, Duke University School of Medicine, Durham, North Carolina, USA.; 5 Duke Molecular Physiology Institute, Duke University, Durham, North Carolina, USA.; 6 Department of Neurology, Duke University Medical Center, Durham, North Carolina, USA.; 7 Department of Surgery, Duke University Medical Center, Durham, North Carolina, USA.

**Keywords:** Pulmonology, Transplantation, Molecular biology

## Abstract

Chronic lung allograft dysfunction (CLAD) is the leading cause of death in lung transplant recipients. CLAD is characterized clinically by a persistent decline in pulmonary function and histologically by the development of airway-centered fibrosis known as bronchiolitis obliterans. There are no approved therapies to treat CLAD, and the mechanisms underlying its development remain poorly understood. We performed single-cell RNA-Seq and spatial transcriptomic analysis of explanted tissues from human lung recipients with CLAD, and we performed independent validation studies to identify an important role of Janus kinase–signal transducer and activator of transcription (JAK-STAT) signaling in airway epithelial cells that contributes to airway-specific alloimmune injury. Specifically, we established that activation of JAK-STAT signaling leads to upregulation of major histocompatibility complex 1 (MHC-I) in airway basal cells, an important airway epithelial progenitor population, which leads to cytotoxic T cell–mediated basal cell death. This study provides mechanistic insight into the cell-to-cell interactions driving airway-centric alloimmune injury in CLAD, suggesting a potentially novel therapeutic strategy for CLAD prevention or treatment.

## Graphical abstract

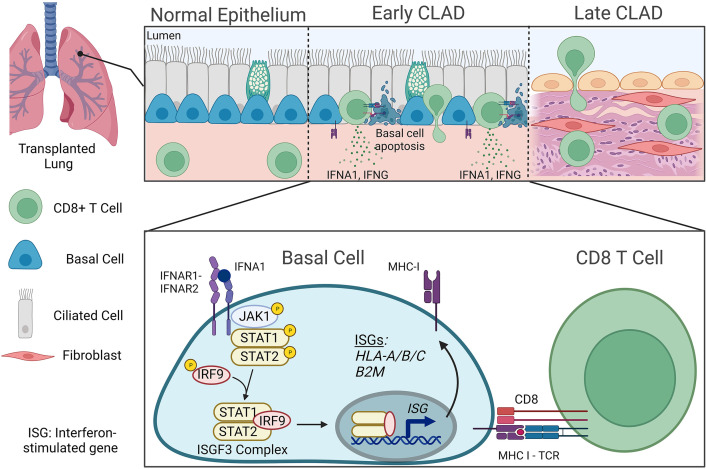

## Introduction

Chronic lung allograft dysfunction (CLAD) is the leading cause of death in lung transplant recipients and contributes to impaired quality of life and increased health care costs (1, 2). CLAD occurs in more than 50% of lung recipients by 5 years after transplant, despite current immunosuppressive strategies. CLAD is characterized clinically by a progressive, irreversible decline in pulmonary function (3) and histologically by bronchiolitis obliterans (BO) in which typical lesions demonstrate obliteration of small airways (4). Importantly, studies suggest BO is a shared histopathological feature of described CLAD phenotypes, which include BO syndrome and restrictive allograft syndrome (4, 5). The precise mechanisms that contribute to CLAD are yet to be fully elucidated, and there are no therapies proven to prevent or treat CLAD (6, 7).

Prior studies suggest that airway epithelial injury is a critical step in the development of CLAD-related airway fibrosis (8–10), and airway epithelial–immune interactions have been explored to identify potential mechanisms contributing to BO development (11–13). Alloimmune T cell reactivity likely plays an important role in the disease, given that the characteristic bronchiolar lesions are rarely found in patients without transplantation and that lymphocytic bronchiolitis has been shown to be an independent risk factor for CLAD (14). Animal lung and tracheal transplant models have identified a possible role for both helper T cells (15) and cytotoxic T cells (16) in the initial alloimmune response against the airways, suggesting that airway epithelial–immune interactions may contribute to airway pathology after transplantation. Such persistent immune cell targeting of the airways may engage further innate and adaptive immune responses and initiate matrix remodeling that result in BO (17, 18). Recent data suggest that current approaches to immune suppression fail to prevent alloimmune targeting of airway epithelial cells, possibly due to the persistence of tissue resident memory CD8^+^ T cells following glucocorticoid therapy (19). There have been few changes in current maintenance or augmented immunosuppressive medications employed since the onset of clinical lung transplantation, and no meaningful improvements have occurred in outcomes for patients with CLAD (20).

A major challenge in developing treatments for BO has been the limited ability to resolve airway cell–specific mechanisms in the spatially heterogeneous disease characteristic of human CLAD airways. Additionally, while there have been advances in preclinical models (8, 21–23), animal models often fail to fully recapitulate human CLAD disease, likely due to distinct airway epithelial cell populations unique to humans and nonhuman primates (24). To overcome these limitations and elucidate specific airway epithelial–immune cell interactions that underlie CLAD development in humans, we performed unbiased single-cell RNA-Seq in lungs explanted from patients undergoing retransplantation for CLAD. Pathway analysis of epithelial cells in CLAD lungs compared with normal controls showed profound gene expression signature changes specific to basal cells, the primary airway epithelial progenitor cell in the lung. Discovery and validation studies involving 10 independent CLAD lungs revealed that activation of Janus kinase–signal transducer and activator of transcription (JAK-STAT) signaling in basal cells leads to upregulation of major histocompatibility complex 1 (MHC-I), a well-established target for cytotoxic CD8^+^ T cells (25). Subsequently, we found enrichment of cytotoxic CD8^+^ T cells in diseased airways and evidence of cytotoxic CD8^+^ T cell–mediated basal cell death. Finally, we leveraged the morphological context provided by spatial transcriptomic analysis to show that this process occurs in airways prior to the development of advanced BO. These data uncover a potentially novel mechanism targeting alloimmune host T cell reactivity to recipient airway basal cells in CLAD and provides a putative therapeutic target for CLAD prevention or treatment distinct from existing posttransplant immunosuppression.

## Results

### Defining the single-cell transcriptome of human CLAD.

We performed single-cell transcriptome profiling from lungs explanted from 4 patients undergoing retransplantation for CLAD and from 3 donor lung controls (Supplemental Table 1; supplemental material available online with this article; https://doi.org/10.1172/jci.insight.167082DS1). Histopathology confirmed the presence of BO in all CLAD cases. Donor control lung tissue was obtained from donor lungs used for transplant that were harvested, preserved, implanted, and then trimmed to fit the recipient chest cavity with excess donor trimming preserved for research. Donor control lung tissue underwent formal pathology evaluation that confirmed the absence of any significant lung disease (Supplemental Table 1).

**Figure 1:**
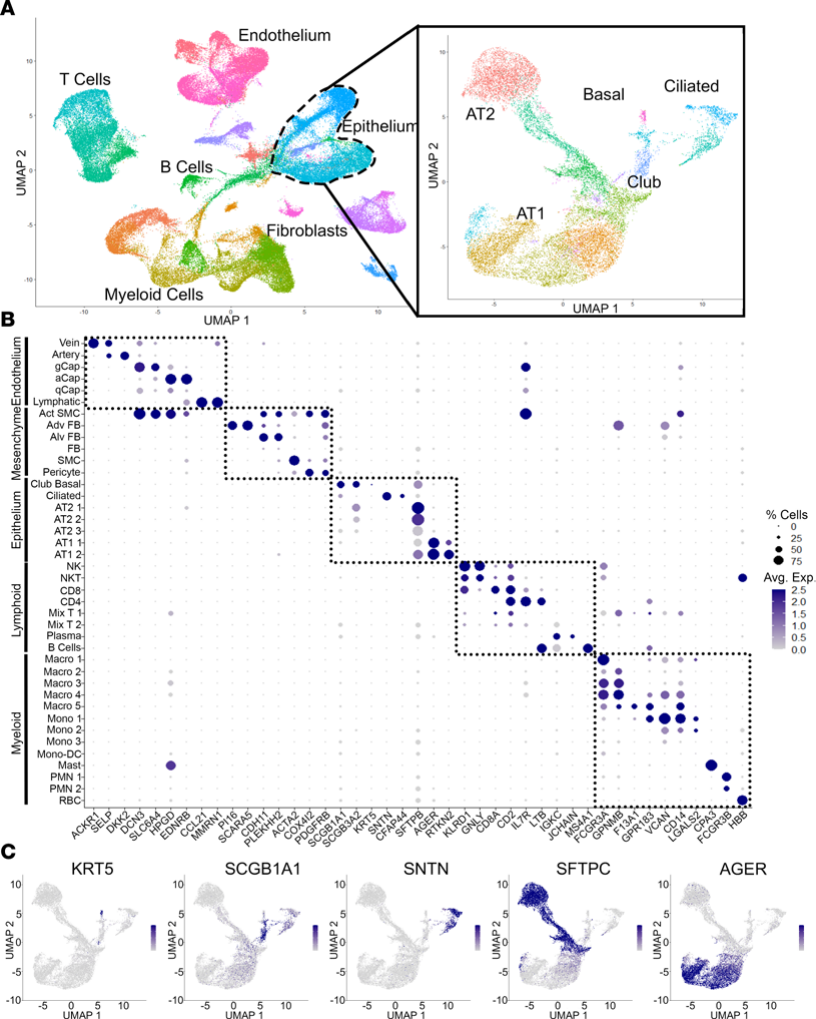
Single-cell RNA-Seq of whole-lung tissue in CLAD. (**A**) UMAP representation of cell clusters from combined 7 samples with epithelial subset analysis showing distinct epithelial cell subpopulations. (**B**) Gene expression dot plot showing unique expression patterns to define 40 lung cell populations in **A**. Complete annotations with cell counts are provided in Supplemental Data File 1. (**C**) Gene expression feature plot showing expression of canonical epithelial cell markers used to define epithelial cell clusters in **A**.

Following data normalization, scaling, and principal component analysis (PCA), we performed uniform manifold approximation and projection (UMAP) that identified 83,456 cells clustered in 40 populations with representation of most known expected lung cell types, including endothelial, epithelial, immune, and mesenchymal lineages (Figure 1, Supplemental Figure 1, and Supplemental Data File 1). Subpopulations of cells were identified based on prior published single-cell lung data sets (26–28).

We next performed subcluster analysis of the 7 identified epithelial cell clusters to better characterize the epithelial cell types affected in this airway-centric disease. This subcluster analysis identified distinct airway epithelial cell populations differentiated by expression of genes, including *KRT5*, *SCGB1A1*, *SCGB3A2*, *FOXJ1*, *SNTN*, *AGER*, and *SFTPC* (Figure 1 and Supplemental Figure 2A). These populations include basal cells, ciliated cells, differentiating ciliated cells, secretory cells, alveolar epithelial cells (AT1 and AT2 cells), and respiratory airway secretory (RAS) cells, as previously described (27, 28).

### MHC-I and IFN gene sets are upregulated in CLAD lung basal cells.

**Figure 2:**
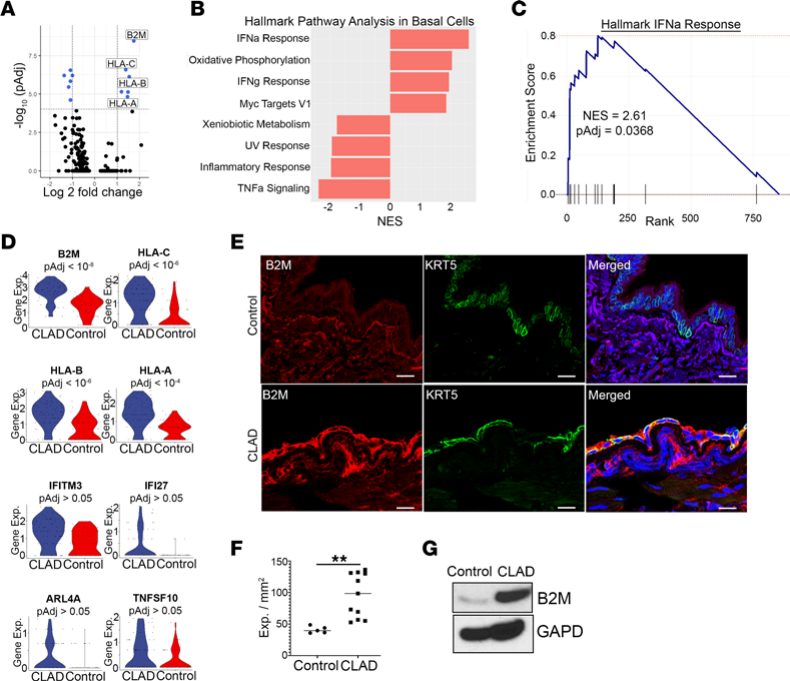
Transcriptional targets of IFN response, including MHC-I expression, are upregulated in CLAD basal cells compared with donor controls. (**A**) Volcano plot of gene expression changes in CLAD (*n* = 4) compared with control (*n* = 3) basal cells showing upregulation of MHC-I–expressing genes. (**B**) Hallmark pathway analysis of 855 preranked genes (Supplemental Data File 2) showing pathways significantly different (*P*_adj_ < 0.05) in CLAD compared with control basal cells. (**C**) GSEA plot showing enrichment of IFN-α and IFN-γ response genes in CLAD basal cells. (**D**) Violin plots demonstrating upregulation of IFN response genes in CLAD compared with control basal cells. (**E**) Immunofluorescence staining of control (top) and CLAD (bottom) airways showing increased expression of MHC-I (red), as measured by antibody staining of B2M, in KRT5^+^ basal cells (green) from CLAD airway tissue compared with control. Yellow in merged panel shows colocalization of MHC-I expression present in CLAD but not donor controls. Scale bar: 30 μm. (**F**) Quantification of MHC-I expression in basal cells in every airway across 3 CLAD and 3 control samples. (**G**) Immunoblotting showing increased protein expression of B2M in epithelial-enriched protein lysates from CLAD compared with control airway tissue. Differential gene expression (**A**–**D**) was performed using the Wilcoxon rank sum test with Bonferroni correction to generate adjusted *P* values. Statistical analysis for protein quantification (**F**) was performed using the unpaired parametric *t* test. Data are shown as mean ± SEM. **P < 0.01.

Given that basal cells are the primary airway progenitor cell population in the lung and that loss of airway basal cells has been reported in experimental transplant BO (8), we hypothesized that the transcriptome of airway basal cells would be altered in human CLAD. As such, we performed differential gene expression analysis in CLAD compared with control cells within the basal cell population. Strikingly, we found that 4 of the most upregulated transcripts were genes transcribing MHC-I proteins, including β2 microglobulin (B2M) and human leukocyte antigens (HLA), HLA-A, HLA-B, HLA-C, and HLA-F (Figure 2A, Supplemental Figure 2, and Supplemental Data File 2). MHC-I gene expression was the most upregulated in basal cells compared with any other cell type from the single-cell data set (Supplemental Figure 2B). Pathway analysis of all 24,690 genes within basal cells showed significant enrichment of IFN-α and IFN-γ gene sets in CLAD (Figure 2, B–D). Importantly, IFN signaling is known to regulate MHC-I gene expression through binding of IFN-regulatory factor (IRF) members on IFN-stimulated response elements (ISRE) in the proximal promoter regions of MHC-I class genes (29, 30).

To confirm this finding of increased MHC-I expression in CLAD lung epithelial cells and to localize MHC-I expression in basal cells specifically, we first performed immunoblotting of epithelial cell–enriched protein lysates following negative selection by CD45 and CD31 bead sorting of single-cell digests of CLAD and donor control lung airway segments. Western blot analysis showed an increase in B2M protein expression in CLAD compared with donor control airway epithelial cell–enriched lysates (Figure 2G). Next, we performed immunofluorescence in frozen CLAD and donor control lung tissues for MHC-I–associated proteins using the commonly employed pan–HLA-ABC monoclonal antibody W6/32, which binds to the N-terminal domain on B2M (31). We found a significant increase in MHC-I protein expression specifically in CLAD airway basal cells compared with control, in concordance with the single-cell transcript data (Figure 2, E and F, and Supplemental Figure 2D).

### JAK-STAT signaling is activated in CLAD lung basal cells.

**Figure 3:**
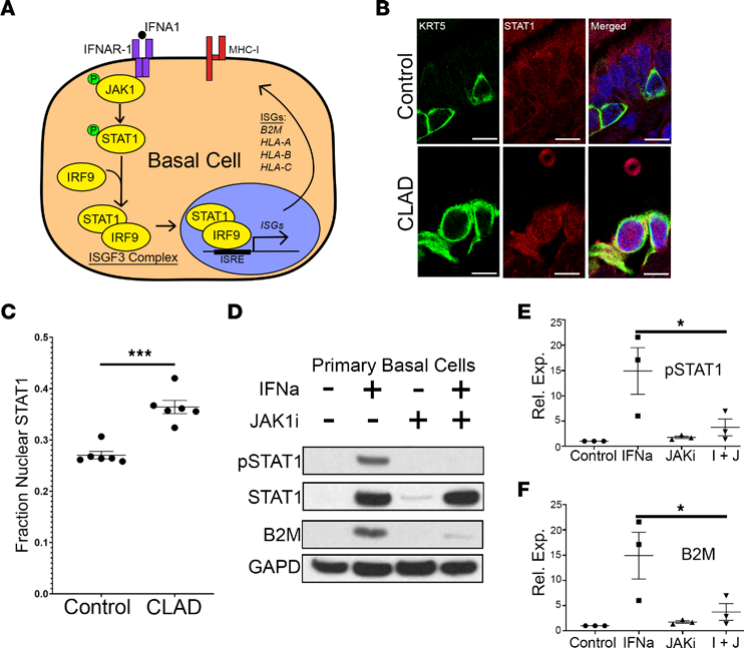
Activation of JAK-STAT pathway in CLAD basal cells mediates overexpression of MHC-I. (**A**) Schematic showing JAK-STAT activation leads to nuclear localization of STAT1 where it binds to IFN-sensitive response element (ISRE) leading to upregulation of IFN stimulating genes (ISGs), including MHC-I–expressing genes (*B2M*, *HLA-A*, *HLA-B*, *HLA-C*). (**B**) Immunofluorescence shows increased nuclear localization for STAT1 (red) in CLAD compared with control basal cells (KRT5^+^, green) (*n* = 6 CLAD cases and 6 controls). DAPI, blue. Scale bar: 10 μm. (**C**) Quantification of nuclear localization of STAT1 in CLAD compared with control basal cells. (**D**) Immunoblotting for phospho-STAT1, STAT1, B2M, and GAPD in primary basal cells isolated from donor controls shows activation of JAK1-STAT1 signaling and upregulation of MHC-I following stimulation with IFNA1 (100 ng/μL). This response is partially rescued with cotreatment with the JAK1 inhibitor, brepocitinib (0.5 μM). (**E** and **F**) Quantification showing a significant decrease in IFN-mediated activation of JAK-STAT pathway in cells treated with a JAK1 inhibitor (*n* = 3 biological replicates). Statistical analysis for immunofluorescence experiments was performed using the unpaired parametric *t* test, while analysis for Western blots was performed using the 1-way ANOVA (*P* < 0.05) with post hoc Tukey test. Data are shown as mean ± SEM. *P < 0.05, ***P < 0.001.

Type I and II IFNs bind to their associated cell surface receptor and mediate important immune cell responses through JAK and its downstream family of transcription factors, STATs, as part of the JAK-STAT signaling cascade (Figure 3A). Activation of STAT1 through its phosphorylation leads to its nuclear localization, where it acts as a transcription cofactor for genes regulating immune response (32). IFN-mediated JAK-STAT activation has previously been shown to play an important role in lung immune response to SARS-CoV-2 (33) and influenza (34) infections but has not been described in the context of lung allograft dysfunction.

To evaluate whether JAK-STAT signaling is active in CLAD lung basal cells, we performed immunofluorescence staining on formalin-fixed, paraffin-embedded (FFPE) tissue sections from CLAD and donor control lungs. Control lungs were obtained from 2 sources: 3 donor control samples, as previously described, and 3 lung transplant controls without CLAD (Supplemental Table 1). Lung transplant controls were obtained at the time of surgical lung biopsy for non-CLAD indications, with formal pathology review that showed no significant lung airway pathology (Supplemental Table 1). In using these samples as additional controls, we were able to perform comparisons between groups of lung transplant recipients receiving similar immunosuppression.

Immunofluorescence showed a significant increase in STAT1 nuclear localization, a surrogate marker for its activation, in basal cells within CLAD compared with control airways (Figure 3, B and C, and Supplemental Figure 3, A and B). There was no significant difference in STAT1 nuclear localization in donor control and lung transplant control airways (Supplemental Figure 3C). Notably, there was no significant increase in nuclear localization of STAT1 in airway ciliated or secretory cells in CLAD (Supplemental Figure 3), suggesting that JAK-STAT activation may be specific to basal cells.

### JAK-STAT activation is required for increased MHC-I expression in airway basal cells following IFN stimulation.

We next evaluated whether JAK-STAT activation is required for the increase in MHC-I expression observed in CLAD basal cells compared with control lung basal cells. We isolated basal cells from control lung airways as previously described (35). Primary passage 1 basal cells were then treated with and without IFNA1 (100 ng/μL) in the presence or absence of the JAK1-specific inhibitor, brepocitinib (0.5 μM), to test whether JAK-STAT activation is necessary and/or sufficient to induce increased MHC-I expression. Protein lysates were harvested 24 hours after treatment, and immunoblotting for the MHC-I protein, B2M, and phospho-STAT1 was performed. Western blot analysis showed a significant increase in MHC-I protein expression following IFNA1 stimulation that was abrogated by treatment with the JAK1-specific inhibitor (Figure 3, D–F). These data suggest that JAK-STAT activation is necessary and sufficient for IFNA1-mediated MHC-I expression in basal epithelial cells.

### Cytotoxic CD8^+^ T cells are enriched in CLAD lungs.

Next, we explored the potential functional consequences of JAK-STAT activation and MHC-I upregulation on lung epithelial–immune interactions. MHC-I presentation of peptides recognized as nonself in the context of infection, cancer, or alloimmunity can result in cell targeting by cytotoxic CD8^+^ T cells (25, 36, 37). We hypothesized that JAK-STAT–mediated upregulation of MHC-I in lung allograft epithelial cells contributes to alloimmune airway injury by recipient cytotoxic CD8^+^ T cells.

**Figure 4:**
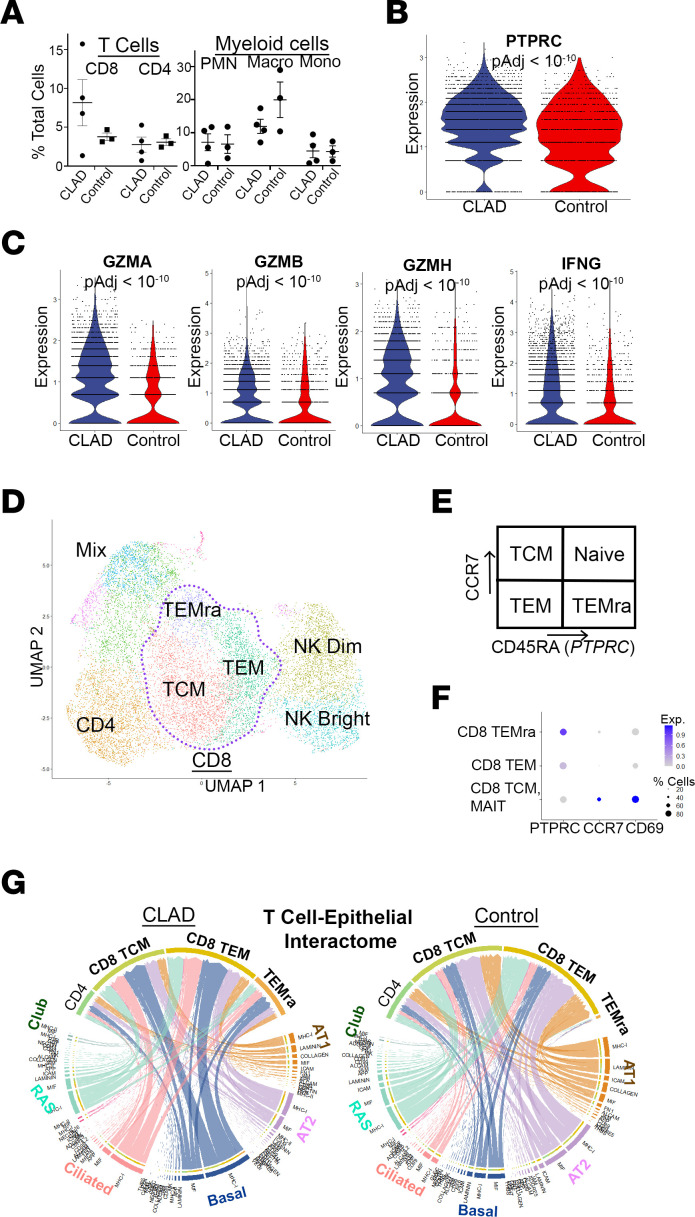
Enrichment of activated and mature cytotoxic CD8^+^ T cells in CLAD lung compared with donor control. (**A**) Cell count analysis of single-cell RNA-Seq data set shows enrichment of total CD8 cell populations (no statistically significant difference). (**B**) Violin plot of *PTPRC* (CD45RA) gene expression shows increased maturation of CD8^+^ T cells in CLAD lung. (**C**) Violin plots demonstrating increased expression of activation markers (GZMA, GZMB, GZMH, IFNG) in CLAD CD8^+^ T cells compared with donor control. (**D**) Subcluster analysis of the T cell population from the single-cell RNA-Seq data set. (**E**) Schematic of markers (*PTPRC*, *CCR7*) of T cell maturation. (**F**) Gene expression dot plot used to annotate subclusters identified in **D**. (**G**) CellChat analysis of predicted T cell–epithelial cell interactions in CLAD (left) compared with control (right) lung cells. The chord diagram shows a predicted increase in CD8^+^ cell, and especially CD8^+^ TEMRA cell, interactions with basal and ciliated airway epithelial cells. Statistical analysis for cell counts (**A**) was performed using unpaired parametric *t* tests. Differential gene expression (**B** and **C**) was performed using the Wilcoxon rank sum test with Bonferroni correction to generate adjusted *P* values. Data are shown as mean ± SEM.

First, we examined the CLAD and donor control single-cell transcriptome data to compare overall numbers of immune cell subsets in CLAD lungs compared with donor control. We found enrichment of CD8^+^ T cells but not CD4^+^ T cells, monocytes, neutrophils, or macrophages in CLAD compared with control lungs (Figure 4A; Supplemental Figure 1, C and D; and Supplemental Data File 1). Interestingly, gene expression analysis of the CD8^+^ T cell subpopulation showed a significant increase in the T cell maturation marker, *PTPRC*, which encodes CD45RA (Figure 4B). Prior data suggest that this maturation process may play an important role in the alloimmune CD8^+^ T cell response that persists following glucocorticoid therapy in the context of acute lung rejection in human lung transplant recipients (19). We also identified an increase in gene expression of granzymes (GZMA/B) and IFN (IFNG), the functional secreted proteins of cytotoxic cells, in the CD8^+^ T cell subpopulation in CLAD compared with control lungs, suggesting an increase in activated cytotoxic CD8^+^ T cells in CLAD (Figure 4C).

Second, we performed subcluster analysis of the T cell populations to identify specific subsets of interest, including effector memory, central memory, and mature effector memory CD8^+^ T cells (TEM, TCM, and TEMRA, respectively), using well-described markers for these cell states including CD45RA, CCR7, and CD69 (Figure 4, D–F). To evaluate for functionally relevant gene expression profile changes within these CD8^+^ T cell subsets and identify their plausible interactions with airway epithelial cell populations, we performed CellChat analysis of the single-cell RNA-Seq data set (38). The CellChat database includes receptors, ligands, and cofactors of more than 2,000 heteromeric molecular complexes essential in known cell-to-cell interactions. It is a tool that quantitatively infers and analyzes these intercellular communication networks in single-cell RNA-Seq data sets and allows for predictions of signaling inputs and outputs for each subpopulation of cells. CellChat analysis showed an increase in predicted CD8^+^ TEM cell, particularly CD8^+^ TEMRA cell, to basal cell interaction specifically via MHC-I signaling (Figure 4G and Supplemental Figure 4). The specific CellChat receptor-ligand gene expression interactions are shown in Supplemental Figure 4. These data suggest that there is increased donor basal cell–recipient cytotoxic T cell alloimmune response in CLAD airways.

### Cytotoxic CD8^+^ T cells target basal cells for cell death in early CLAD–affected airways.

Prior studies of explant lungs have noted the spatial and temporal heterogeneity of disease in airways of patients with CLAD, with a range of epithelial injury, inflammation, and fibrosis, present across different airways within the same patient (39). Distinct airways millimeters apart can demonstrate the full spectrum of disease from normal to BO (Supplemental Figure 5). We sought to evaluate whether there is increased cytotoxic CD8^+^ T cell–mediated basal cell death in CLAD airways both compared with control airways and by comparisons across the histological spectrum of disease, from less affected airways that may represent an earlier disease stage to more severely affected airways reflective of advanced disease. Early CLAD–affected airways were identified as those with completely intact airway epithelium by staining for airway cell markers with minimal fibrosis, whereas advanced CLAD-affected airways were identified as those with complete fibrotic airway obliteration (Supplemental Figures 5–7).

**Figure 5:**
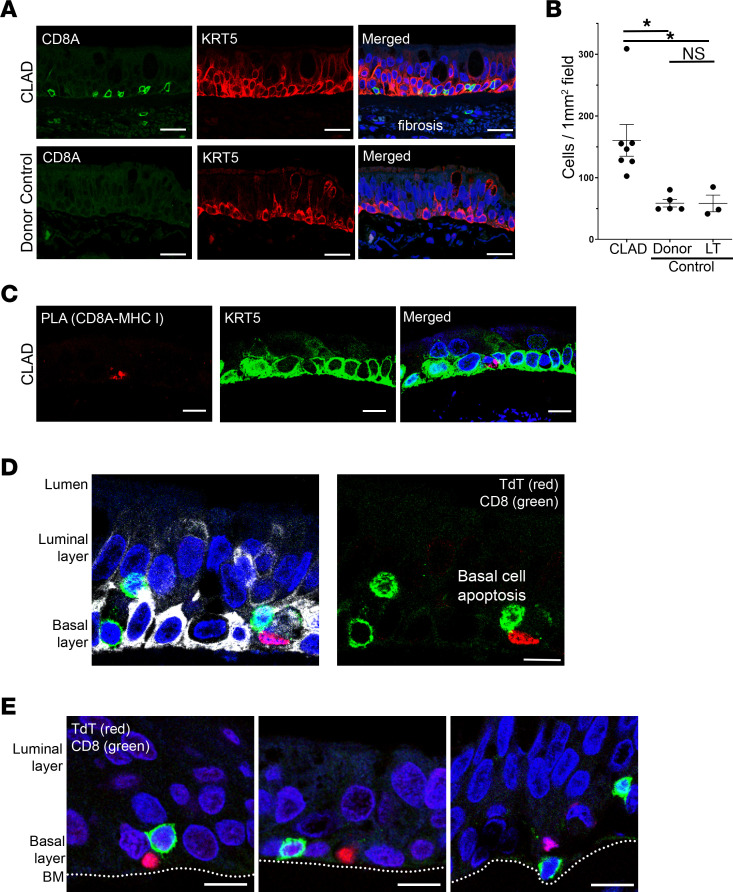
CD8^+^ T cells colocalize with KRT5^+^ basal cells and induce apoptosis in CLAD airways. (**A**) Immunofluorescence staining for CD8^+^ T cells (CD8A, green) shows increased colocalization with basal cells (KRT5, red) in CLAD airways (top) compared with control airways (bottom) (*n* = 7 CLAD cases and 8 controls). Extensive fibrosis was notable in CLAD airways as indicated by nonspecific staining. Scale bar: 20 μm. (**B**) Quantification of immunofluorescence showing significantly increased CD8^+^ cells in CLAD airways compared with donor and lung transplant (LT) control tissue. There was no significant difference in CD8^+^ cell airway localization between the 2 control groups. (**C**) Proximity ligation assay with probes targeting CD8A and B2M (as a surrogate for MHC-I) identified a putative ligand-receptor interaction between CD8^+^ T cells and basal cells (KRT5) in CLAD airways. (**D** and **E**) TUNEL staining (TdT, red) revealed multiple cases of basal cell (KRT5, white) apoptosis in direct association with CD8^+^ T cells (CD8A, green) in CLAD airways that were not observed in control airways. Airway basement membrane (BM) is annotated for reference in **E**. Scale bar: 10 μm. Statistical analysis for immunofluorescence quantification (**B**) was performed using the 1-way ANOVA (*P* < 0.05) with post hoc Tukey test. Data are shown as mean ± SEM. *P < 0.05.

First, we performed immunofluorescence staining for CD8A (CD8^+^ T cell marker) and keratin 5 (basal cell marker; KRT5) and found a significant increase in colocalization of CD8^+^ T cells with basal cells in CLAD compared with control donor and transplant lungs (Figure 5, A and B, and Supplemental Figure 6). There was no significant difference in the presence of CD8^+^ T cells in donor control airways compared with lung transplant control airways (Figure 5B). Remarkably, confocal imaging showed that 95% of all CD8^+^ cells within early CLAD–affected airways localize to the basement membrane and directly replace basal cells at their expected location (Figure 5, A–C, and Supplemental Figure 6), in comparison with advanced-stage lesions that demonstrate complete obliteration of the airway with diffuse lymphocytic infiltration (Supplemental Figure 6).

Next, to identify whether there is a direct ligand-receptor–mediated cell-to-cell interaction between CD8^+^ T cells and basal cells, we performed a proximity ligation assay. Using probes targeting CD8A and B2M followed by counter-staining for the basal cell marker, KRT5, we found ligand-receptor interactions between CD8^+^ T cells and basal cells in CLAD but not control airways (Figure 5C). These findings validate the CellChat-based inferences based on the single cell data set (Figure 4) and suggest that not only are CD8^+^ T cells enriched in CLAD airway epithelium, but they are actively engaged specifically with basal cells.

Finally, we evaluated the functional consequences of CD8^+^ T cell–basal interactions in CLAD airway epithelium. We performed TUNEL staining for apoptosis with costaining for CD8A and KRT5, and we identified apoptosis of basal cells in direct association with CD8^+^ T cells in CLAD airways but not in control airways (Figure 5, C–E, and Supplemental Figure 6). Furthermore, while advanced CLAD-affected airways showed diffuse cell death of many cell types (Supplemental Figure 6C), early CLAD–affected airways showed basal cell–specific cell death (Figure 5, C–E). These data suggest a mechanism of BO that begins with CD8^+^ T cell–mediated basal cell death in early CLAD–affected airways. The depletion of this important progenitor cell type provides a putative mechanism for impaired airway repair that leads to the development of advanced fibrosis in CLAD airways.

### Spatial transcriptomic analysis identifies enrichment of CD8^+^ TEM cells in early CLAD–affected airways.

**Figure 6:**
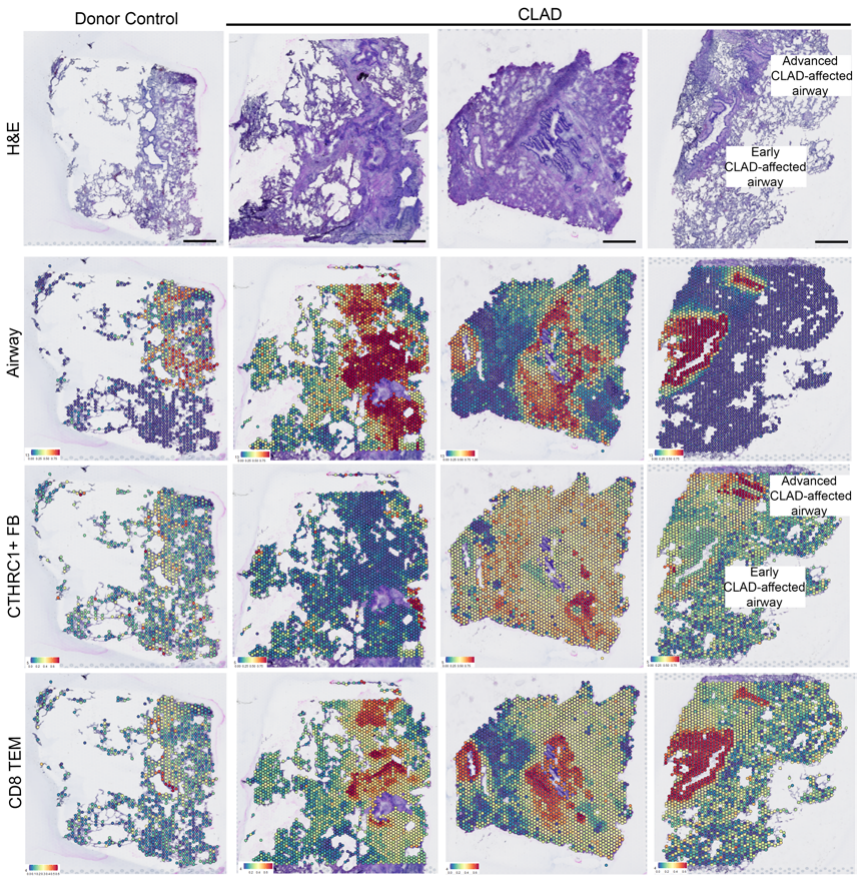
Enrichment of CD8^+^ TEM cells in airway epithelial cells in diseased CLAD airway epithelium. H&E staining (top panel) for control (left) and 3 CLAD samples (right) on which spatial transcriptomic analysis was performed. Airway secretory cells, CTHRC1^+^ fibroblasts, and CD8^+^ TEM cells defined by single-cell RNA-Seq data set were mapped onto control (left panel) and CLAD (right 3 panels) spatial transcriptomic data sets. The results show enrichment of CD8^+^ TEM cells in early CLAD–affected airways.

To explore the specific cell-to-cell interactions driving CD8^+^ T cell recruitment to CLAD epithelium and, subsequently, basal cell death, we performed spatial transcriptomic analysis on tissue sections from lungs explanted from 3 patients undergoing retransplantation for CLAD (including 1 used previously in single-cell analysis and 2 independent of previous studies) and 1 donor lung control (Figure 6 and Supplemental Table 1). Visium spatial gene expression slides allow for mapping of the whole transcriptome within 50 μm barcoded spots at a 1- to 10-cell resolution (40). This provides morphological context encompassing the spatial and temporal heterogeneity of CLAD based on correlating H&E sections and mapping of airway cell and fibroblast subpopulations (Figure 6). To characterize cell populations within each barcoded spot, we mapped cell populations defined by the single-cell RNA-Seq data set (Figure 1) onto all spatial transcriptomic samples using an anchor-based integration workflow (41). Interestingly, and independently validating our single-cell and protein expression results, we found colocalization of CD8^+^ TEM cells specifically — and not CD8^+^ TCM, NK, or CD4^+^ T cell populations — within CLAD airway epithelium (Figure 6 and Supplemental Figure 7). Notably, colocalization was present even in early CLAD–affected airways with intact epithelium, minimal fibrosis, and fewer activated, CTHRC1^+^ fibroblasts (42). These findings independently support the single-cell and immunofluorescence data suggesting that CD8^+^ TEM cells mediate basal cell death in early CLAD–affected airways.

## Discussion

Survival after lung transplantation is limited primarily by the development of CLAD, a shared histological feature of which is BO. Though several studies have investigated directed therapies to prevent CLAD or slow the progression of established CLAD, to date, there remain no effective treatments. Here we used data from whole-lung single-cell RNA-Seq and spatial transcriptomic analysis of human CLAD tissues to show that activation of JAK-STAT signaling leads to upregulation of MHC-I in airway basal cells and contributes to cytotoxic CD8^+^ T cell–mediated basal cell death.

Single-cell sequencing and spatial transcriptomic analysis offers an unbiased investigative approach into understanding the mechanisms of airway epithelial cell injury in CLAD. Strengths of this study include the direct analysis of human CLAD lung tissue samples and independent validation of our findings using single-cell RNA-Seq, spatial transcriptomics, immunofluorescence of tissue blocks, and immunoblotting in fresh tissue across a total of 11 independent CLAD explanted lungs. In addition, the use of control lung tissue from donor lungs that were trimmed prior to use for transplantation, rather than discarded donors, as well as controls from lung transplant controls to account for concurrent immunosuppression add to the study rigor. Importantly, we observed increased recruitment of CD8^+^ T cells to basal cells in CLAD but not CLAD-free transplant patients on similar immunosuppression regimens consistent with our observations when comparing CLAD airways to the donor controls. We found no significant differences in the CD8^+^ T cells present between the donor control and transplant control samples, demonstrating that this effect is specific to airways in CLAD. Furthermore, we leveraged the morphological context provided by spatial transcriptomic analysis and validation immunofluorescence studies to understand mechanisms of basal cell death in early CLAD–affected airways. Our combined approach of single-cell and spatial transcriptomics analysis demonstrates the richness of these techniques to uncover disease mechanisms.

We focused on lung airway epithelial to immune interactions because CLAD is defined by airway-centered immune responses progressing to airway obliteration. We found a common pathway — JAK-STAT activation — that leads to MHC-I upregulation specifically in basal cells and promotes ligand-receptor interaction between host CD8^+^ cytotoxic T cells and basal cells that leads to basal cell death in CLAD airways. Identification of JAK-STAT pathway activation within basal cells was based on 2 independent pathway analysis databases — gene set enrichment analysis (GSEA) and CellChat analysis. Recognizing the limitations associated with RNA-Seq analysis with limited sample size, an additional strength of our study is the inclusion of protein validation studies performed using immunofluorescence and Western blot analysis of additional and independent samples of CLAD and control lung tissue that confirm these results.

Our data suggest that upregulation of MHC-I specifically in basal cells likely plays an important role for mediating the airway-centered fibrosis in CLAD. The MHC-I consists of a single heavy α-chain expressed from 1 of 3 distinct gene regions — *HLA-A*, *HLA-B*, and *HLA-C* — in complex with the invariant light chain, *B2M* (43). Although MHC-I is expressed on the surface of all nucleated cells, its expression is tightly regulated and can be modulated in inflammation and cancer (29, 32). Following transplantation, MHC antigens from donor tissue are recognized by the recipient immune system, generating an alloimmune response (44). Prior literature has identified viral infection in patients following transplant as an independent risk factor for developing CLAD, and it has been hypothesized that prior viral infection may “prime” recipient airway epithelial cells for targeting by host T cells (45–47).

Our data suggest important cell-to-cell interactions between cytotoxic T cells and airway epithelial cells in driving IFN-mediated JAK-STAT activation that leads to MHC-I upregulation and T cell–mediated basal cell death. Upregulation of both type I and II IFNs could be driven by viral infection or other noxious exposures in the transplant allograft (48, 49). Further studies will be needed to better define the specific mechanisms that lead to IFN upregulation and recruitment of CD8^+^ T cells to the airway epithelium in CLAD. Prior studies have shown that upregulation of MHC-I leads to targeting by cytotoxic CD8^+^ T cells for cell death (25, 37, 50), and downregulation of MHC-I has been associated with tumor cell and viral mechanisms to evade the innate immune response (25, 36). Given these data, the JAK-STAT pathway represents a potentially novel and promising therapeutic target to prevent or treat CLAD.

Although upregulation of MHC I might be expected in the allograft, it is interesting to note that, in our single-cell data set, the upregulation of MHC-I is specific to a small number of cell types, most notably basal epithelial cells (Supplemental Figure 2). Given the importance of basal cells as airway progenitors following injury, progressive depletion of airway stem cells via cytotoxic CD8^+^ T cell–mediated cell death suggests a mechanism that impairs normal lung regeneration and may contribute to airway-centered fibrosis characteristic of CLAD. This is supported by findings that cytotoxic CD8^+^ T cell–mediated basal cell death is present in early CLAD–affected airways with intact epithelium and minimal fibrosis. These data also propose a mechanism that explains previous findings that basal cells specifically are depleted in a ferret model of lung transplant CLAD and human CLAD (8).

We recognize that a potential limitation of our study is the use of CLAD tissue from patients undergoing pulmonary retransplantation. Notably, studies in other lung diseases, such as idiopathic pulmonary fibrosis (IPF), have used single-cell RNA-Seq of explant lung tissue to provide insights in disease pathogenesis (42, 51, 52). Prospective evaluation of lung tissue from patients following lung transplantation as a patient progresses from a healthy allograft state to CLAD would be even more useful. However, transbronchial lung biopsies, which are often performed as part of allograft monitoring, provide mostly alveolated as opposed to airway tissue and would not provide adequate tissue for a comprehensive single-cell analysis. In contrast, tissues explanted from patients undergoing retransplantation for CLAD provide adequate tissue and permit the study of the spatial and temporal heterogeneity of CLAD-affected airways (Figure 6). Our findings of CLAD-affected airways with differing severity are consistent with prior studies that have described the histological spectrum of epithelial injury, inflammation and fibrosis present in CLAD lungs (39). In leveraging the morphological context provided by our immunofluorescence and spatial transcriptomic data, we found that JAK-STAT activation and CD8^+^ T cell localization to airways is present in early CLAD–affected airways, as confirmed by intact epithelium and minimal fibrosis (Figure 6 and Supplemental Figure 6).

In conclusion, we demonstrate that JAK-STAT signaling mediates MHC-I upregulation in airway basal cells in CLAD, resulting in cytotoxic CD8^+^ T cell–mediated basal cell death. Our results demonstrate that inhibition of JAK signaling diminishes basal cell MHC-I expression, illustrating the value of JAK inhibition as a putative treatment modality in CLAD. Current strategies for reducing the host immune response to donor tissue include maintenance immunosuppression regimens such as calcineurin pathway inhibitors downstream of T cell receptor signaling, corticosteroids, and cell cycle inhibitors. However, these existing approaches do not directly modulate JAK-STAT signaling. With the emergence of pan- and selective JAK inhibitors in the treatment of inflammatory conditions, including alloimmune-mediated conditions such as graft-versus-host disease, our data provide strong rationale for further study of JAK inhibition in human lung transplant recipients.

## Methods

### Human lung sample preparation for single-cell sequencing.

A 2 ***×*** 2 cm segment of lung tissue was isolated at least 1 cm from the pleura and minced using a razor blade. Tissue was digested at 37°C using Multi Tissue Dissociation Kit 1 (Miltenyi Biotec, 130-110-201) for 30 minutes. Mechanical digestion was performed 3 times using gentleMACS dissociator (Miltenyi Biotec, 130-093-235). The cell suspension was spun down at 300*g* for 10 minutes, and cells were treated with RBC lysis buffer (MilliporeSigma, R7757). The cell suspension was filtered using a 70 μm filter, spun down at 300*g* for 10 minutes, and resuspended in PBS with 0.04% BSA. Single cells were prepared for cDNA amplification and library preparation using Single Cell 3′ Reagent Kits v2 (10x Genomics) at the Molecular Genomics Core facility at the Duke Molecular and Physiology Institute (DMPI). The cDNA libraries were sequenced on the NovaSeq 6000 SP and S1 platforms by the Duke Center for Genomic and Computational Biology core facility.

### Human lung single-cell sequencing data processing.

The primary analytical pipeline for single-cell sequencing analysis followed the recommended protocols from 10x Genomics. Briefly, Cell Ranger v5.0.1 was used to demultiplex raw FASTQ files, align the reads to the 10x GRCh38 reference transcriptome, and generate gene expression matrices for all single cells in each sample. Further downstream analysis was performed in R v4.1.0. The R package Seurat v4.1.0 (41) was used for quality control and further downstream analysis on the feature-barcode matrices produced by Cell Ranger. The top 3,000 genes were selected based on variance using the Seurat implementation FindVariableFeatures. These genes were then centered and scaled using Seurat’s implementation ScaleData and percent mitochondrial genes as a variable to regress out (R code in parentheses) (percent_mt = 0.25). Scaled data were subsequently used for principal components analysis (npcs = 42), and cell cluster analysis was performed using Seurat’s FindNeighbors (dims = 1:42) and FindClusters (resolution = 1.2) functions. Visualization was performed using UMAP. Cell counts, mean reads per cell, and median genes per cell for each sample are provided in Supplemental Figure 1. Differential gene expression analysis was performed using the Seurat’s FindMarkers function, which uses a Wilcoxon rank sum test and Bonferroni correction to generate adjusted *P* values for each gene. Pathway analysis was performed using the fast GSEA (fgsea) package. CellChat (38) analysis was performed using default settings and filtered for cell clusters containing a minimum of 10 cells.

### Visium spatial transcriptomics.

H&E staining of OCT embedded CLAD and donor control samples was performed to identify airway tissues with definitive CLAD morphology and normal controls. Three CLAD and 1 donor control samples embedded in OCT were chosen to proceed to Visium (Supplemental Table 1). Briefly, 10 μm sections were cut from frozen OCT blocks and mounted onto Visium spatial gene expression slides and processed according to the manufacturer’s protocol (10x Genomics). Tissue sections were permeabilized immediately to capture RNA molecules onto barcoded spots on the slide. Libraries were then prepared and sequenced on the NovaSeq 6000 SP platform by the Duke Center for Genomic and Computational Biology core facility.

Fiducial 50 μm spots on the H&E image were manually aligned using Loupe browser v5.0.1 (10x Genomics). Space Ranger software (10x Genomics) was used to process raw FASTQ files, align the reads to the 10x GRCh38 reference transcriptome, and generate gene expression matrices for all single cells in each sample. Further downstream analysis of the spot UMI table and reference H&E section was performed in R using Seurat v4.1.0. Data were normalized using SCTransform and spatial gene expression was visualized using Seurat’s SpatialFeaturePlot function. Integration of single-cell RNA-Seq data was performed using the FindTransferAnchors function with predicted positions of cells visualized using the SpatialFeaturePlot function.

### In vitro culture and assays.

Primary human bronchial epithelial cells were isolated as previously described (35). Single-cell digests underwent negative selection with CD45^+^ (Miltenyi Biotec, 130-045-081) and CD31^+^ (Miltenyi Biotec, 130-091-935) bead sorting. In total, 300,000 cells of this epithelial enriched population were directly lysed for Western blot analysis, as shown in Figure 2G. The remaining cells were plated in culture as previously described (35). A total of 30,000 passage 1 cells was plated into each well of 6.5 mm polyester inserts containing 0.4 μm pores (MilliporeSigma, CLS3470) coated with Type IV human placenta collagen (MilliporeSigma, C7521). In total, 100 μL of air liquid interface (ALI) medium was added to the insert and 600 μL of ALI medium was added to the well at the time of plating. Twenty-four hours after plating, cells were treated with IFN-α (Abcam, ab48750) at a concentration of 100 ng/μL with and without the JAK1 inhibitor, PF-06700841 (MilliporeSigma, PZ0345), at a concentration of 0.5 μM for 24 hours. For immunoblotting, cells were lysed in RIPA buffer with protease and phosphatase inhibitors (Santa Cruz Biotechnology, sc-24948). Protein was quantified (Thermo Fisher Scientific, 23225), and equal amounts of protein was separated by gel electrophoresis (Thermo Fisher Scientific, NP0322BOX) and transferred onto PVDF membrane (Thermo Fisher Scientific, IB24001) using Thermo Fisher Scientific iBlot2 Dry Blotting System. Membranes were probed with antibodies to STAT1 (Cell Signaling Technology, 9172), pSTAT1 (Cell Signaling Technology, 9167), B2M (Cell Signaling Technology, 12851), and GAPDH (Santa Cruz Biotechnology, sc-32233) overnight at 4°C; thoroughly washed with TBS with 0.05% Tween-20; and then incubated with secondary HRP-tagged antibodies for 1 hour at room temperature. Blots were incubated using chemiluminescent reagents (Thermo Fisher Scientific, 34580) and developed using x-ray film (VWR, PI34090).

### Immunofluorescence.

In total, 5 μm FFPE lung tissue sections were deparaffinized, rehydrated, and heat inactivated (BioCare Medical Decloaking Chamber) in citrate buffer (pH 6) for antigen retrieval. Sections were blocked in 3% goat serum in TBS with 0.05% Tween-20 for 1 hour. Sections were incubated with primary antibodies to KRT5 (BioLegend, 905901), STAT1 (Cell Signaling Technology, 9172), CD8A (MilliporeSigma, 108M-9), acetylated α-tubulin (MilliporeSigma, T7451), and SCGB1A1 (R&D Systems, MAB4218) overnight at 4°C in a humidified chamber. Sections were then washed with TBS-T, followed by incubation with secondary antibody in blocking solution for 1 hour at room temperature. Sections were then washed with TBS-T and mounted using aqueous mounting media containing DAPI counterstain. Five-micrometer OCT-frozen sections were required for staining with the B2M antibody (BioLegend, 311402), as this antibody did not recognize the appropriate epitope on FFPE sections. TUNEL staining was performed as directed using a TUNEL in situ apoptosis kit (Elabscience, E-CK-A322) prior to costaining with antibodies. The Proximity Ligation Assay was performed per recommended manufacturer’s protocol (MilliporeSigma, DUO92105) with probes targeting B2M (Abcam, ab215889) and CD8A (Abcam, ab217344). Cell imaging was performed on the Leica SP8 confocal microscope at the Duke Light Microscope Core Facility. Quantification was performed across all cells of all identified airways in each section using particle analysis function in Fiji software. For representative high-resolution images, linear scale adjustments were applied to STAT1, KRT5, CD8A, and B2M. Representative stitched confocal images of entire airways are provided in Supplemental Figures 2, 3, 5, and 6.

### Data and materials availability.

All data associated with this study are present in the paper or the supplemental materials. The raw single-cell RNA-Seq data sets are available at the NCBI Gene Expression Omnibus (GEO) (accession no. GSE224210; access token, stwzykkubnkttmd).

### Statistics.

The number of patient samples for each experiment is indicated in the figure legends. Data aggregation and statistical analyses for immunofluorescence and Western blot studies were performed using GraphPad Prism (v9.4.0). All graphs are represented as mean ± SEM, and a 2-tailed *P* value of less than 0.05 was considered significant. Power calculations were used to determine the necessary sample size for all immunofluorescence experiments based on effect size and results observed in the single-cell RNA-Seq data. Differences in continuous variables between 2 groups was assessed using the unpaired parametric 2-tailed *t* test or Wilcoxon rank test. Comparisons between more than 2 groups were assessed using 1-way ANOVA. Post hoc Tukey testing for multiple-comparison tests was only performed when statistical significance (*P* < 0.05) was identified from the ANOVA. Fisher’s exact test was used for analysis of T cells in association with basal cells based on the contingency table shown in Supplemental Figure 6. All quantification for immunofluorescence studies was performed using stitched images for every airway for every sample, and representative stitched samples are provided in Supplemental Figures 2, 3, and 6. Representative high-resolution confocal images for each group are indicated in the main figures.

### Study approval.

The research objective of this study was to use unbiased single-cell transcriptomic analysis to identify mechanisms contributing to the BO phenotype characterized in CLAD. The sample size and biological replicates were determined by primary sample availability — a limitation, given the paucity of lung retransplant cases. CLAD cases were limited to those with definitive clinical and pathological diagnosis, while control samples were limited to those without significant pathological evidence for airway disease (Supplemental Table 1). Upon identification of JAK-STAT signaling as a putative target, validation studies with immunofluorescence staining for protein were performed on samples used for transcriptomic studies as well as additional and independent CLAD and control lung tissue samples. Sample size needed for statistical significance in validation studies was determined by power calculations as described in the statistics section. The number of samples and biological replicates are specified in figure legends. To establish a causal relationship between IFN signaling, JAK-STAT activation, and MHC-I expression, we treated primary human basal cells from donor controls with IFN-α and a JAK1 inhibitor. These experiments were performed in 3 biological replicates. Outliers are included. Informed consent was obtained from all patients, and the study was approved by the Duke IRB Pro00104727, Pro00100758, and Pro00108982.

## Author contributions

Conceptualization was contributed by AK, JLT, and SMP. Methodology was contributed by AK, JLT, FLK, AN, ZJ, SGG, VJ, and SMP. Investigation was contributed by AK, JLT, FLK, AN, VJ, and SMP. Visualization was contributed by AK and VJ. Funding acquisition was contributed by AK, JLT, and SMP. Project administration was contributed by JLT, FLK, and SMP. Supervision was contributed by JLT, SGG, ZJ, KJW, and SMP. Writing of the original draft was contributed by AK and SMP. Review and editing of the manuscript were contributed by AK, JLT, FLK, AN, ZJ, JV, SGG, KJW, and SMP.

## Supplementary Material

Supplemental data

Supplemental data set 1

Supplemental data set 2

## Version 1 03/22/2023

Electronic publication
