# Supplementary material for: JAK-STAT activation contributes to cytotoxic T cell–mediated basal cell death in human chronic lung allograft dysfunction
Source: JCI Insight. 2023 Mar 22;8(6):e167082. doi: 10.1172/jci.insight.167082 (PMC10070100; doi:10.1172/jci.insight.167082)
Supplement: Supplemental data [file jciinsight-8-167082-s017.pdf]

Supplementary Information

CLAD Whole Lung Patient Characteristics

Age	Sex	CLAD Stage	Histology Report	Native Disease	Time to Transplant (days)	Calcineurin Inhibitor	Third Agent	Augmentation last 6 mths
62	M	Stage 3	A0B0C1	Pulmonary veno-occlusive disease	709	Cyclosporine	MMF	Rituxan, Belatacept
60	F	Stage 3	A1C1	Idiopathic pulmonary fibrosis	3589	Tacrolimus	MMF	None
26	F	Stage 4	A2B2RC1	Cystic fibrosis	472	Tacrolimus	Azathioprine	Solu Medrol
63	M	Stage 3	A3B1RC1	Idiopathic pulmonary fibrosis	1555	Tacrolimus	None	None

Donor Control Patient Characteristics

Age	Sex	CLAD Stage	Cause of Death	Histology Report
53	F	Stage 0	Anoxia	Mild emphysematous changes with patchy bronchiolitis and mild pulmonary hypertensive arteriopathic changes
61	M	Stage 0	Stroke	Patchy exudative acute lung injury with mild chronic interstitial pneumonia
15	M	Stage 0	Trauma	No histopathologic diagnosis

Lung Transplant Control Patient Characteristics

Age	Sex	CLAD Stage	Histology Report	VATS Indication	Time to Biopsy (days)	Calcineurin Inhibitor	Third Agent	Augmentation last 6 mths
61	M	Stage 0	A0B0C0	Pleural effusion	552	Tacrolimus	Azathioprine	Solu Medrol
68	M	Stage 0	A2B0C0	Recurrent exudative effusion	302	Cyclosporine	MMF	None
61	M	Stage 0	A1B1RC0	Exudative effusion	273	Tacrolimus	None	Belatacept, RATG

Spatial Transcriptomic Data Patient Characteristics

Age	Sex	Tissue Type	Histology Report
26	F	CLAD	A2B2RC1; constrictive bronchiolitis with high grade lymphocytic bronchiolitis
27	F	CLAD	A1B1C1; chronic allograft rejection with features of constrictive bronchiolitis
48	F	CLAD	A0B1RC1; constrictive bronchiolitis with low grade lymphocytic bronchiolitis without perivascular inflammation
38	M	Donor control	Lung tissue with mild emphysema. No evidence of acute infection or malignancy.

Supplementary Table 1: Patient characteristics. Patient characteristics for whole lung samples used in single cell RNA sequencing and spatial transcriptomic studies as well as lung transplant control patients used in protein validation studies. The table for transplant controls includes the indication for surgical biopsy via video-assisted thoracoscopic surgery (VATS).

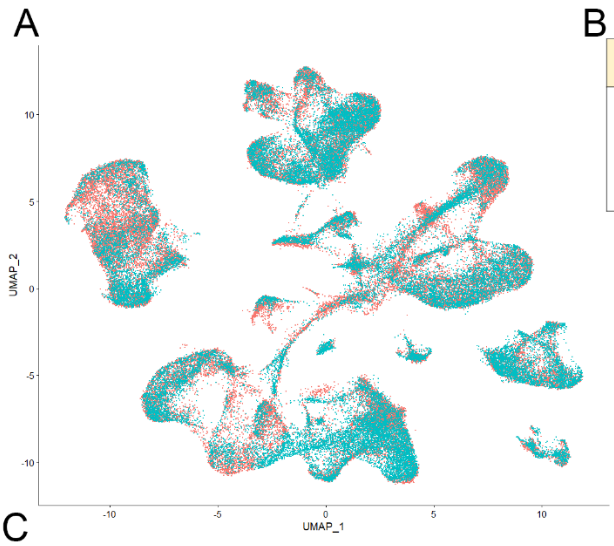

B

	21-013	20-469	18-577	21-361	20-469 control	21-287 control	21-361 control
# Cells	17,880	20,848	6,889	17,675	13,170	10,935	12,473
Mean Reads / Cell	55,749	53,172	133,345	47,645	74,122	84,986	65,802
Median Genes / Cell	865	1,030	1,663	779	1,636	1,683	1,276

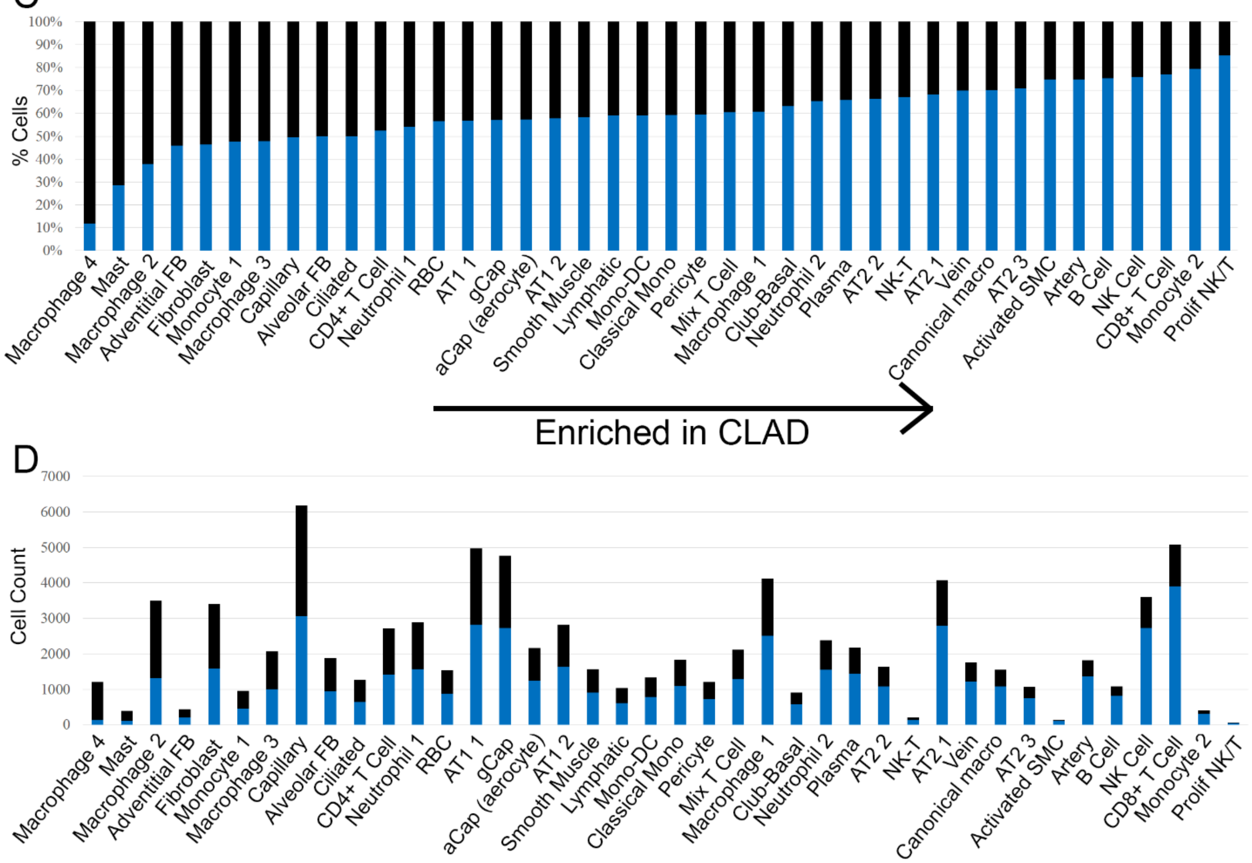

Supplementary Figure 1: Cell count by cluster analysis for CLAD and donor control RNA sequencing data. A. UMAP representation of combined four CLAD patient samples (orange) and three donor control samples (green). B. Raw cell count and read count for each sample. C. CLAD (blue) compared to control (black) cell representation for each cell cluster showing cell types enriched in CLAD lungs compared to control lungs. D. Raw cell count by cluster analysis of four CLAD patient samples (blue) and three donor control samples (black). Complete annotations of cell types are provided in Supplementary Data 1.

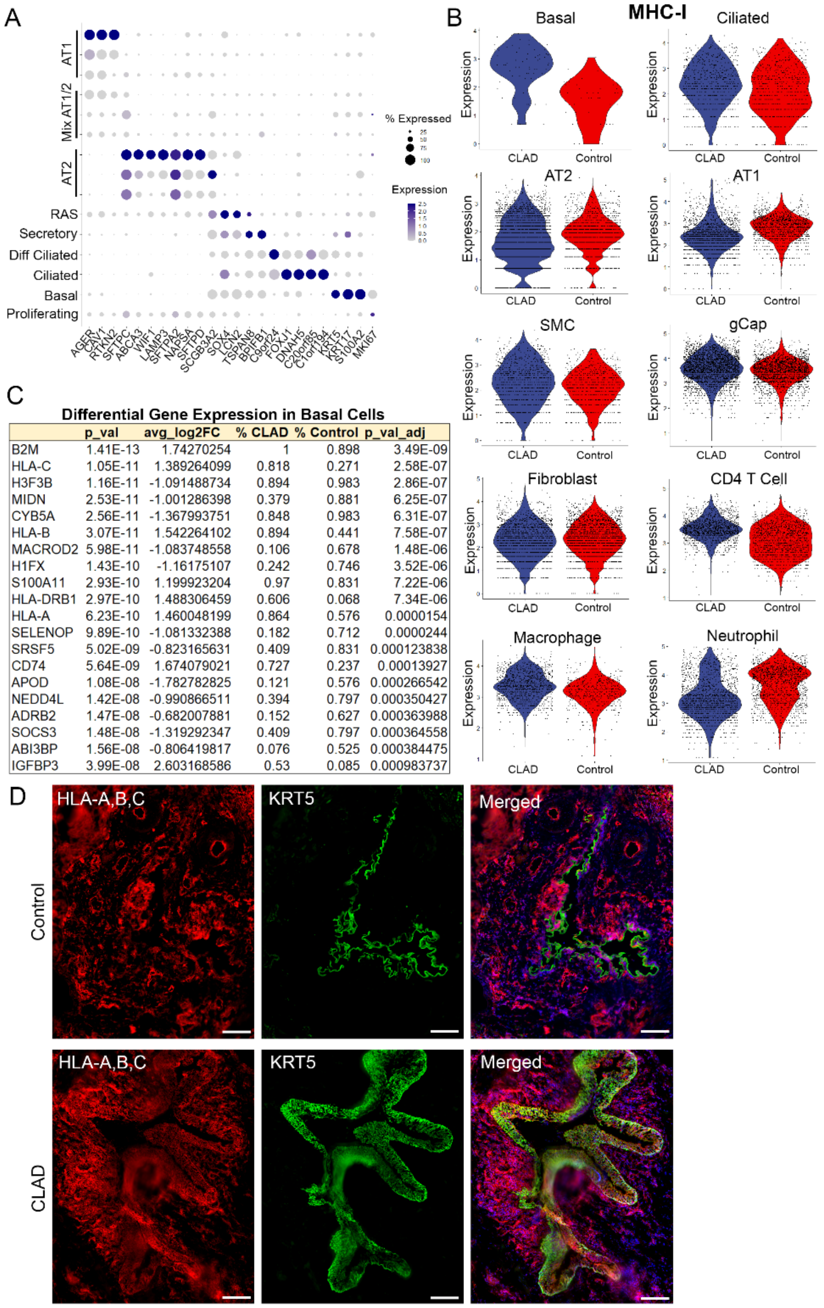

Differential Gene Expression in Basal Cells

	p_val	avg_log2FC	% CLAD	% Control	p_val_adj
B2M	1.41E-13	1.74270254	1	0.898	3.49E-09
HLA-C	1.05E-11	1.389264099	0.818	0.271	2.58E-07
H3F3B	1.16E-11	-1.091488734	0.894	0.983	2.86E-07
MIDN	2.53E-11	-1.001286398	0.379	0.881	6.25E-07
CYB5A	2.56E-11	-1.367993751	0.848	0.983	6.31E-07
HLA-B	3.07E-11	1.542264102	0.894	0.441	7.58E-07
MACROD2	5.98E-11	-1.083748558	0.106	0.678	1.48E-06
H1FX	1.43E-10	-1.16175107	0.242	0.746	3.52E-06
S100A11	2.93E-10	1.199923204	0.97	0.831	7.22E-06
HLA-DRB1	2.97E-10	1.488306459	0.606	0.068	7.34E-06
HLA-A	6.23E-10	1.460048199	0.864	0.576	0.0000154
SELENOP	9.89E-10	-1.081332388	0.182	0.712	0.0000244
SRSF5	5.02E-09	-0.823165631	0.409	0.831	0.000123838
CD74	5.64E-09	1.674079021	0.727	0.237	0.00013927
APOD	1.08E-08	-1.782782825	0.121	0.576	0.000266542
NEDD4L	1.42E-08	-0.990866511	0.394	0.797	0.000350427
ADRB2	1.47E-08	-0.682007881	0.152	0.627	0.000363988
SOCS3	1.48E-08	-1.319292347	0.409	0.797	0.000364558
ABI3BP	1.56E-08	-0.806419817	0.076	0.525	0.000384475
IGFBP3	3.99E-08	2.603168586	0.53	0.085	0.000983737

Supplementary Figure 2: Upregulation of MHC-I expression in CLAD compared to control basal cells. A. Gene expression dot plot showing unique expression patterns defining epithelial cell sub-clusters in Figure 1B. B. Gene expression of B2M in CLAD and control cells showing upregulation of B2M is specific to basal cells, ciliated cells, macrophages, and CD4+ T cells. C. List of top 20 genes (by adjusted p-value) following differential gene expression analysis in CLAD compared to donor control basal cells. Statistical analysis was performed using Wilcoxon rank sum test with Bonferroni correction. Full gene list is provided in Supplementary Date File 2. D. Stitched 20x objective immunofluorescence imaging of representative whole airway in CLAD and donor control lungs showing low level of MHC-I expression (red) in control airway (green = Keratin 5) and upregulation in diseased CLAD airways. Scale bar = 250 µm.

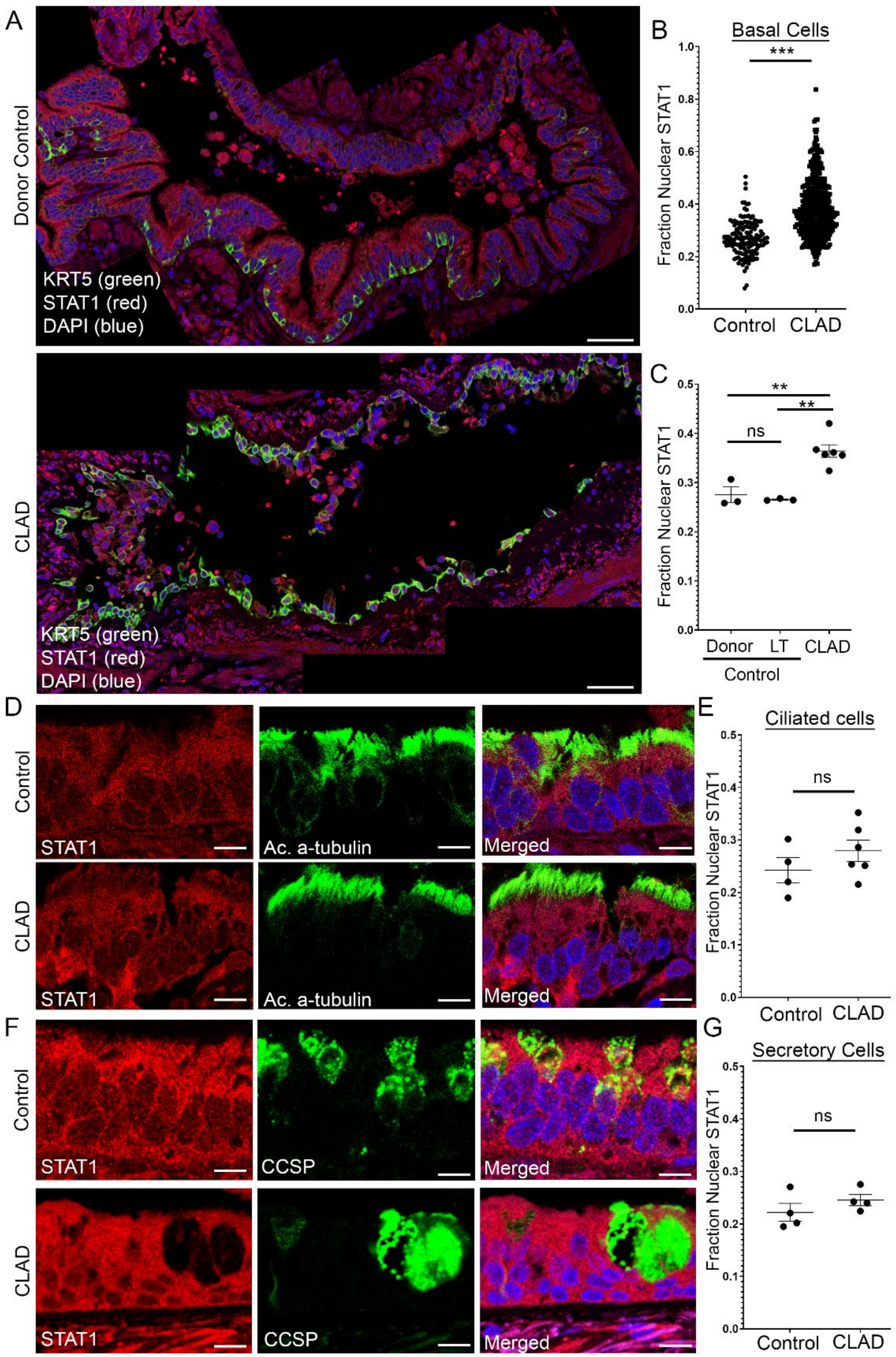

Supplementary Figure 3: Increased nuclear localization of STAT1 in CLAD basal cells but not CLAD ciliated and secretory cells. A. Stitched 40x objective confocal images of immunofluorescent staining for KRT5 (green), STAT1 (red), and DAPI (blue) in CLAD and control airways of similar size. Extensive fibrosis and epithelial damage are present in the CLAD airway. Scale bar = 50µm. B. Quantification of the fraction of nuclear STAT1 across all KRT5+

cells in a single 5μm section from 6 CLAD and 6 control samples. C. Quantification of fraction of nuclear STAT1 in CLAD samples (n=6) compared to lung transplant (LT) control (n=3) and donor control (n=3) showing no significant difference in nuclear STAT1 expression in the two control groups. D-G. Immunofluorescence with quantification showed no significant change in nuclear localization of STAT1 in CLAD ciliated (D-E) or secretory cells (F-G) compared to control. Scale bar = 10μm. Statistical analysis for immunofluorescence experiments was performed using the unpaired parametric t-test (B, E, and G) or one-way ANOVA ($p < 0.05$) with post-hoc Tukey test (C). Graph depicts mean with SEM. ** = $p < 0.01$, *** = $p < 0.001$.

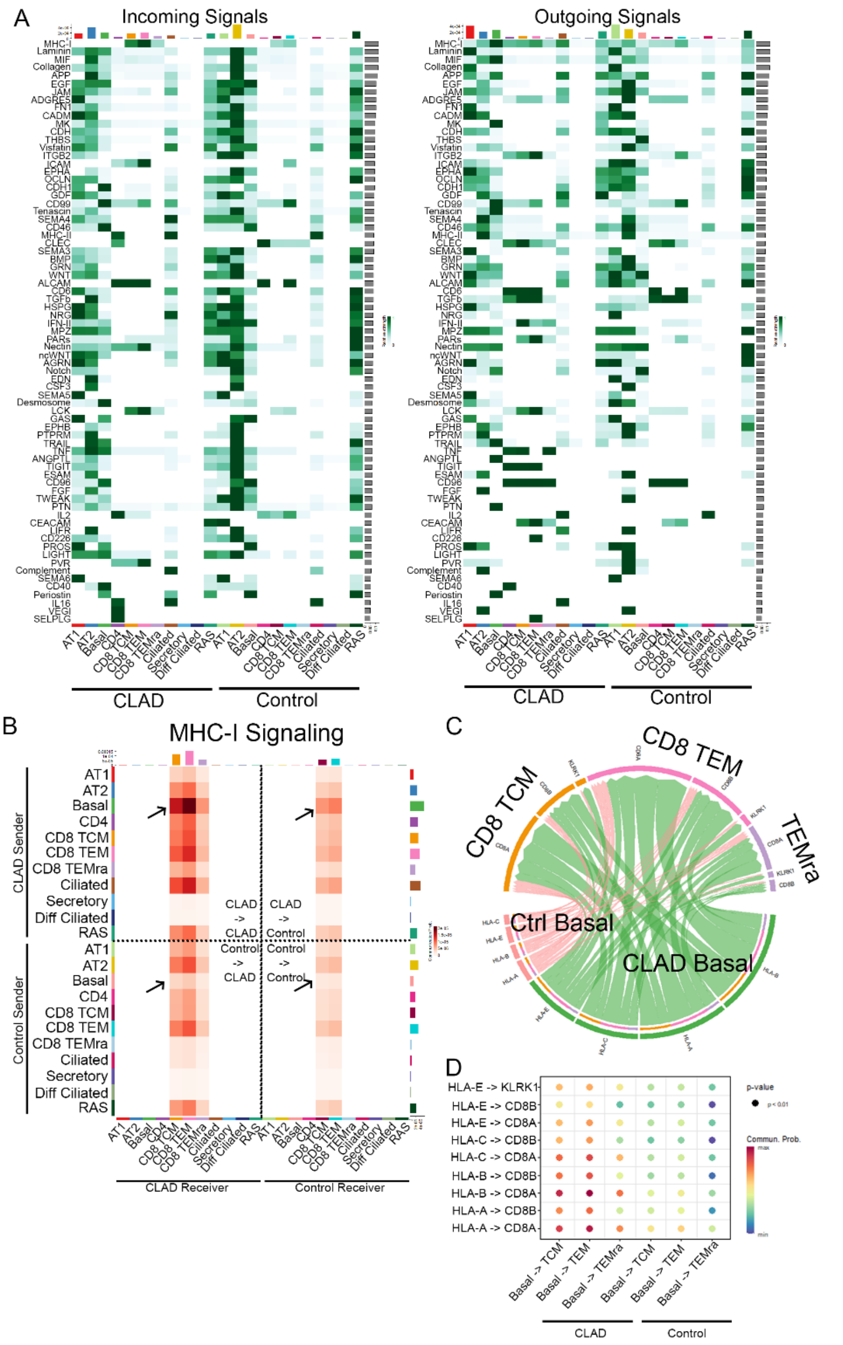

Supplementary Figure 4: Cell Chat analysis predicts increased CLAD basal cell to CD8+ T cell interactions via MHC-I signaling compared to control basal cells. A. Signaling pathways contributing most to outgoing and incoming signals in predicted epithelial-T cell interactions. B. Heatmap of predicted epithelial-T cell interactions via MHC-I signaling shows highest predicted interaction between CLAD basal cells and CLAD CD8+ TEM cells. Arrows show comparison of basal and CD8+ T cell interactions in CLAD and control cells. C. Chord diagram shows a predicted increase in CLAD basal cell compared to control basal cell CD8+ T cell interactions via MHC-I signaling. D. Bubble plot showing all significant predicted MHC-I mediated interactions between basal cells and CD8+ T cells.

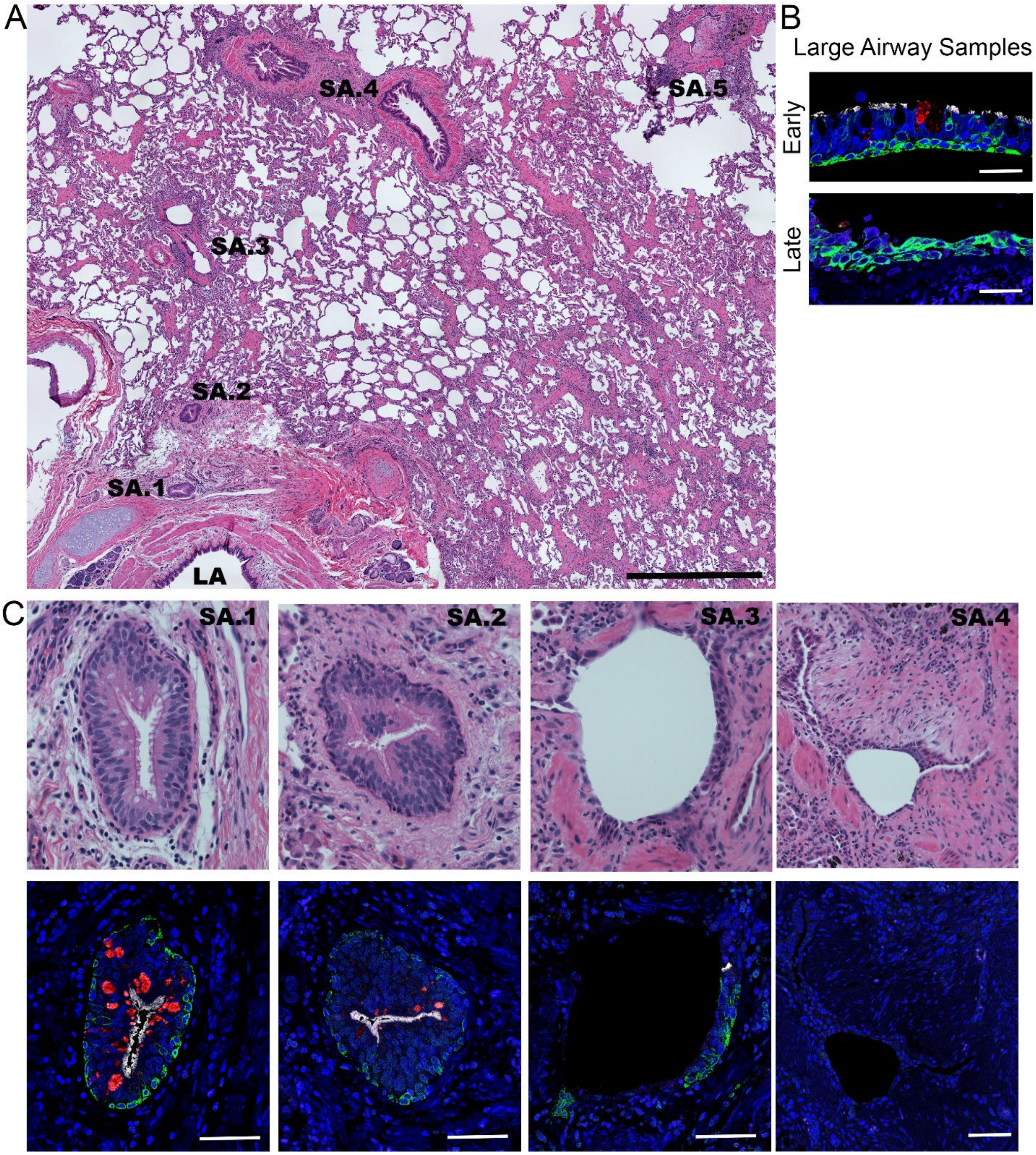

Supplementary Figure 5: Heterogeneity of disease in CLAD. A. H&E staining from a single CLAD patient explant showing large (LA) and small airways (SA) in various stages of disease. Scale bar = 1000um. B. High resolution view of large airways from the same patient sample showing varying degrees of epithelial cell damage from intact epithelium (top) to complete loss of normal epithelial markers in the setting of advanced airway fibrosis (bottom). Keratin 5 = green, SCGB1A1 = red, acetylated alpha-tubulin = white. Scale bar = 50um. C. High resolution H&E staining (top) and immunofluorescence (bottom) from (A) showing four small airways (SA) in different stages of disease as determined by loss of epithelial cells, inflammation, and

peribronchiolar fibrosis. SA.1 provides an example of a normal small airway while SA.4 demonstrates a classic bronchiolitis obliterans lesion. Keratin 5 = green, SCGB1A1 = red, acetylated alpha-tubulin = white. Scale bar = 50um.

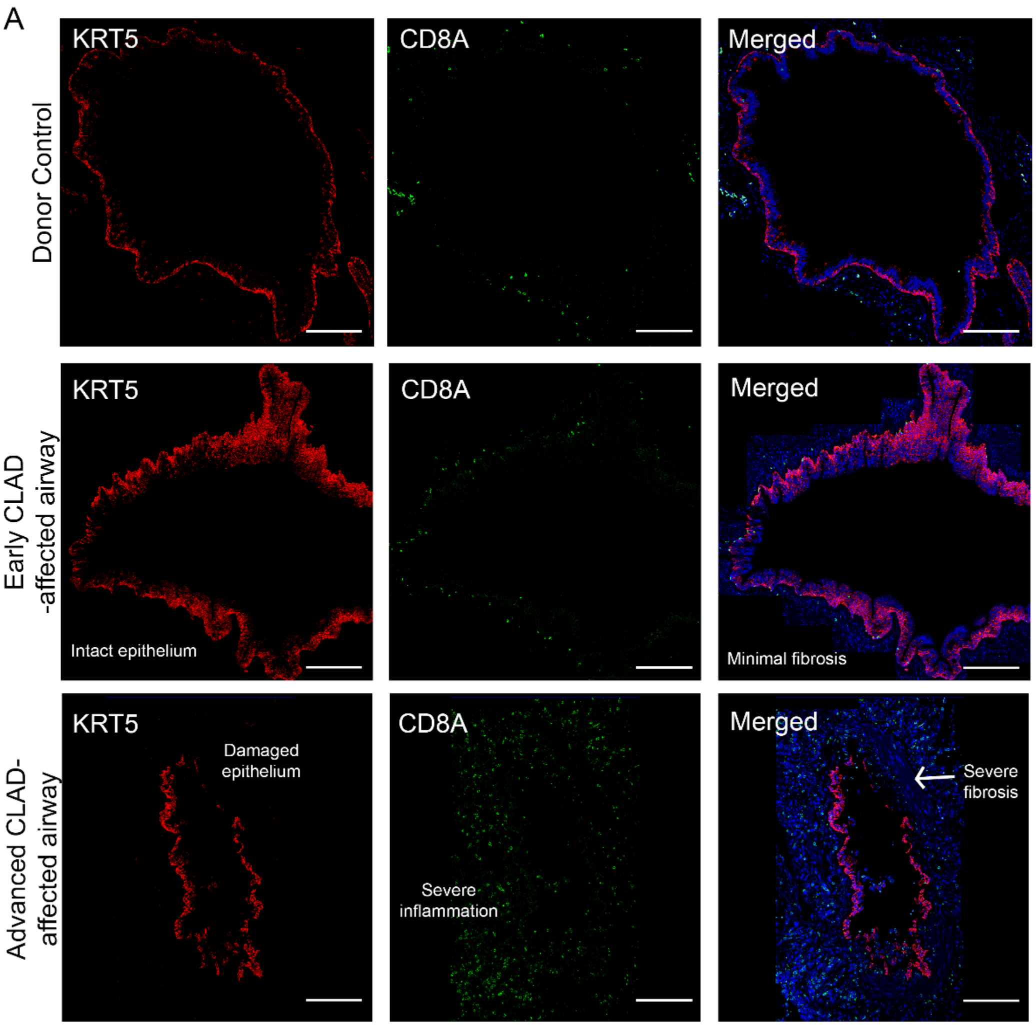

B

			KRT5+ Associated		
			Yes	No	Total
**CD8+ Cells in Airway**	CLAD	Count	334	17	351
		% of CLAD	95.2	4.8	100.0
		% of total	80.9	4.1	85.0
	Control	Count	47	15	62
		% of Control	75.8	24.2	100.0
		% of total	11.4	3.6	15.0

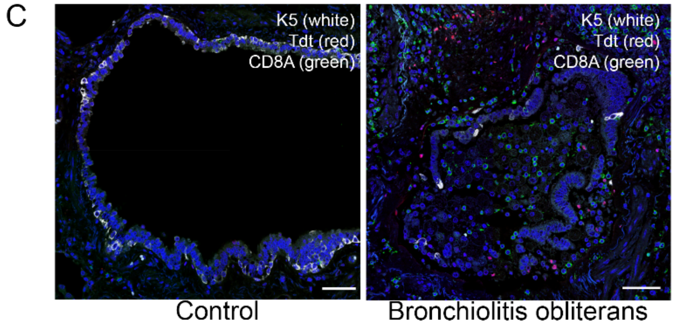

Supplementary Figure 6: Increased co-localization of CD8+ T cells with basal cells in early CLAD-affected airways. A. Stitched confocal images of immunofluorescence staining for CD8A and KRT5 in control (top), early CLAD-affected airways with intact epithelium and minimal fibrosis (middle), and advanced CLAD-affected airways (bottom) shows increased co-

localization of CD8+ T cells with basal cells in early CLAD-affected airways compared to control airways. B. Contingency table of CD8+ T cell counts within early CLAD-affected and control airways in direct association with KRT5+ basal cells. Analysis with Fisher's exact test showed a significant increase ($p < 0.0001$) in CD8+ T cells in direct association with KRT5+ basal cells in early CLAD-affected airways compared to control airways. C. Stitched confocal images of CD8A (green), KRT5 (white), and Tunel staining for apoptosis (red) in CLAD and control airways showing diffuse CD8+ T cell infiltration and apoptosis in a BO lesion in CLAD that were not observed in control airways. Scale bar = 50μm.

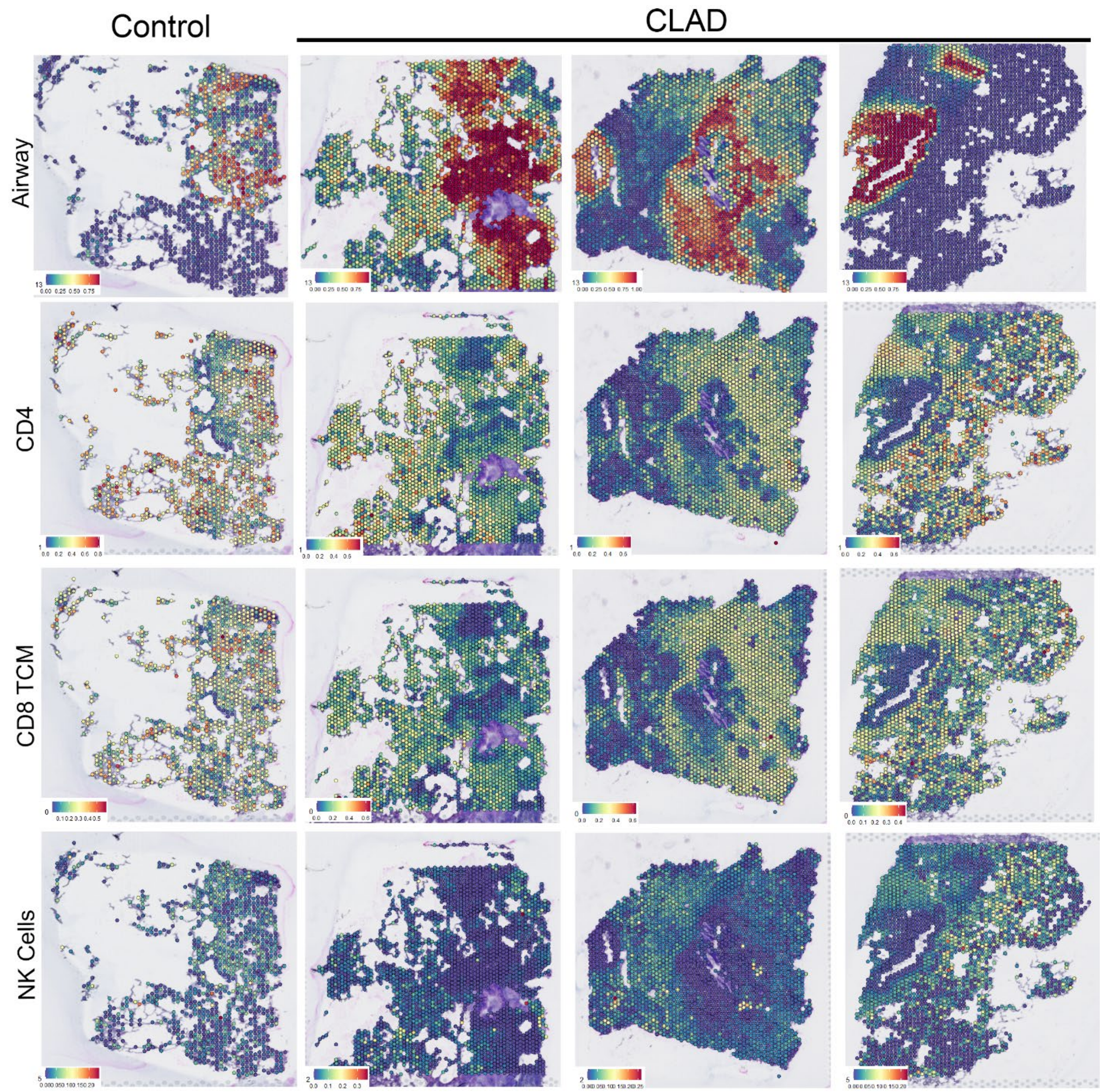

Supplementary Figure 7: Mapping of T cell populations from single cell RNA sequencing dataset onto spatial transcriptomic datasets in CLAD. Mapping of airway secretory cells, CD4 T cells, CD8 TCM cells, and NK cells defined by single cell RNA sequencing dataset (Figure 4) onto control (left panel) and CLAD (right three panels) spatial transcriptomic datasets.
