# Supplementary material for: JAK-STAT activation contributes to cytotoxic T cell–mediated basal cell death in human chronic lung allograft dysfunction
Source: JCI Insight. 2023 Mar 22;8(6):e167082. doi: 10.1172/jci.insight.167082 (PMC10070100; doi:10.1172/jci.insight.167082)
Supplement: Supplemental data set 2 [file jciinsight-8-167082-s019.pdf]

Gene,p_val,avg_log2FC,CLAD,Control,p_val_adj
B2M,1.41E-13,1.74270254,1,0.898,3.49E-09
HLA-C,1.05E-11,1.389264099,0.818,0.271,2.58E-07
H3F3B,1.16E-11,-1.091488734,0.894,0.983,2.86E-07
MIDN,2.53E-11,-1.001286398,0.379,0.881,6.25E-07
CYB5A,2.56E-11,-1.367993751,0.848,0.983,6.31E-07
HLA-B,3.07E-11,1.542264102,0.894,0.441,7.58E-07
MACROD2,5.98E-11,-1.083748558,0.106,0.678,1.48E-06
H1FX,1.43E-10,-1.16175107,0.242,0.746,3.52E-06
S100A11,2.93E-10,1.199923204,0.97,0.831,7.22E-06
HLA-DRB1,2.97E-10,1.488306459,0.606,0.068,7.34E-06
HLA-A,6.23E-10,1.460048199,0.864,0.576,1.54E-05
SELENOP,9.89E-10,-1.081332388,0.182,0.712,2.44E-05
SRSF5,5.02E-09,-0.823165631,0.409,0.831,0.000123838
CD74,5.64E-09,1.674079021,0.727,0.237,0.00013927
APOD,1.08E-08,-1.782782825,0.121,0.576,0.000266542
NEDD4L,1.42E-08,-0.990866511,0.394,0.797,0.000350427
ADRB2,1.47E-08,-0.682007881,0.152,0.627,0.000363988
SOCS3,1.48E-08,-1.319292347,0.409,0.797,0.000364558
ABI3BP,1.56E-08,-0.806419817,0.076,0.525,0.000384475
IGFBP3,3.99E-08,2.603168586,0.53,0.085,0.000983737
SFTPB,4.20E-08,-1.757958328,0.182,0.695,0.001036595
EMP2,4.67E-08,-0.919180767,0.152,0.644,0.001151557
ICAM1,7.04E-08,-1.322742947,0.091,0.508,0.001738613
C6orf132,8.09E-08,-0.512658232,0.045,0.458,0.001996878
CIRBP,8.46E-08,-0.755932101,0.455,0.831,0.002086868
ELF3,8.55E-08,-0.998252338,0.758,0.966,0.002110262
ATP13A4,8.82E-08,-0.676324243,0.061,0.475,0.002175578
CAVIN2,1.18E-07,-0.958217676,0.152,0.559,0.002901064
GPC3,1.45E-07,-1.487626349,0.106,0.508,0.003590385
RGS16,1.47E-07,-0.963205391,0.061,0.458,0.00362118
GOLGA8A,1.64E-07,-0.830545162,0.152,0.593,0.004049023
EPHA2,1.77E-07,-0.685313026,0.152,0.61,0.00435668
SFTA3,1.77E-07,-0.703460373,0.167,0.61,0.004370277
ICAM4,2.35E-07,-0.582082869,0.03,0.407,0.005788143
CXCL2,2.94E-07,-1.344972894,0.455,0.78,0.007244409
CCL20,3.43E-07,-1.419706836,0.061,0.475,0.008453642
ALPL,3.71E-07,-0.626713795,0.061,0.458,0.009155866
CAPN2,4.99E-07,-0.765822394,0.333,0.746,0.012311317
SOD3,6.14E-07,-0.56774343,0.136,0.576,0.015144757
HLA-E,6.52E-07,0.615856509,0.773,0.373,0.016083887
CYP4B1,6.53E-07,-1.048094288,0.364,0.729,0.016128206
DAPK2,6.66E-07,-0.505052816,0.015,0.356,0.016438649
GADD45A,6.93E-07,-0.859924181,0.318,0.729,0.017113734
CIB1,7.11E-07,0.758078581,0.652,0.237,0.017536896
PLAT,8.69E-07,2.075288127,0.409,0.034,0.021446448
SNHG32,9.62E-07,-0.547642224,0.136,0.542,0.023737264
DHCR24,1.01E-06,-0.610341129,0.258,0.678,0.024960328
HOPX,1.13E-06,-1.314702993,0.076,0.458,0.027888059
CLU,1.21E-06,-0.872814801,0.758,0.966,0.029755636

ATOH8,1.21E-06,-0.582082869,0.03,0.373,0.029886524
PMAIP1,1.27E-06,-0.801161355,0.364,0.78,0.031296812
SDC4,1.51E-06,-0.809234655,0.545,0.864,0.037353301
CCND2,1.52E-06,-0.752495923,0.212,0.61,0.037400181
IGFBP2,1.71E-06,-1.032531372,0.909,0.983,0.042223006
KIAA1324,2.21E-06,-0.683703773,0.136,0.542,0.054582485
LTBP3,2.30E-06,-0.445544036,0.045,0.39,0.056758407
SNHG9,2.54E-06,-0.515388025,0.091,0.458,0.062623896
ARAP2,2.69E-06,-0.517231725,0.03,0.356,0.066387251
LAMB3,2.83E-06,-0.766449789,0.561,0.881,0.069725193
SCGB3A1,2.85E-06,-1.037762353,0.273,0.678,0.070245313
PLIN5,2.94E-06,-0.466605652,0.03,0.356,0.072558442
RGCC,3.42E-06,-0.753111342,0.121,0.492,0.084329357
PDLIM2,3.50E-06,-0.581859559,0.076,0.424,0.086496741
TNFAIP3,3.54E-06,-0.759313607,0.121,0.492,0.087308294
SOD2,3.56E-06,-0.759150674,0.47,0.864,0.087767715
MIR22HG,3.89E-06,-0.537260205,0.121,0.492,0.095893037
DUOX1,4.26E-06,-0.567007548,0.121,0.492,0.105220394
PGAM1,4.54E-06,0.504825196,0.424,0.068,0.112103413
CCNL1,4.57E-06,-0.711666624,0.576,0.864,0.112910786
MT2A,5.21E-06,-1.168526066,0.621,0.949,0.128530099
HLA-DRA,5.28E-06,1.275935168,0.561,0.203,0.130296357
ALDH3A2,5.52E-06,-0.548774193,0.273,0.695,0.136291329
FOLR1,5.75E-06,-0.630304079,0.212,0.61,0.141816519
RUNX1,5.76E-06,-0.483679165,0.152,0.542,0.14218897
IFITM3,6.33E-06,1.002784702,0.833,0.644,0.156103744
CNTN4,6.54E-06,-0.346175641,0,0.271,0.161445196
CNKSR3,6.60E-06,-0.709238865,0.182,0.542,0.162808234
ADH1C,7.18E-06,-0.571839353,0.076,0.424,0.17720734
SLPI,8.23E-06,0.994611481,0.788,0.373,0.203177929
RNF145,9.34E-06,-0.62569817,0.606,0.797,0.230606935
SYT8,9.37E-06,-0.946582975,0.136,0.492,0.231266615
HMGB3,9.43E-06,-1.302476095,0.591,0.797,0.232650462
BNIP3,9.55E-06,-0.647177897,0.242,0.61,0.235734725
TUT4,9.94E-06,-0.487737381,0.076,0.407,0.245347109
ST3GAL5,1.01E-05,-0.503857047,0.061,0.39,0.250276316
TSPYL2,1.08E-05,-0.428266045,0.045,0.356,0.267118623
STOM,1.14E-05,-0.898716664,0.333,0.678,0.281248928
PEG10,1.23E-05,-0.704893395,0.061,0.373,0.304177849
GADD45G,1.38E-05,-0.346175641,0.106,0.475,0.339777375
NSG1,1.41E-05,-0.468412408,0.121,0.475,0.348021359
CAPS,1.42E-05,-0.501826512,0.136,0.508,0.351585433
CFLAR,1.61E-05,-0.585890776,0.152,0.508,0.398417501
NFKBIA,1.63E-05,-0.839822975,0.561,0.864,0.402099768
MT1M,1.70E-05,-0.686172029,0.061,0.373,0.420111057
ZBTB16,1.83E-05,-0.653139455,0.045,0.339,0.452362696
APLP2,1.88E-05,-0.637484501,0.667,0.864,0.46287792
HLA-F,1.95E-05,0.483965462,0.364,0.051,0.481511252
TRAF4,2.10E-05,-0.561681677,0.091,0.407,0.517926936
NR1D2,2.50E-05,-0.375154708,0.045,0.339,0.616919035

PSME2,2.73E-05,0.579514662,0.47,0.136,0.674276921
ANOS1,2.75E-05,-0.622724853,0.227,0.576,0.678278735
KMT2C,3.01E-05,-0.378068977,0.03,0.305,0.743177875
KLF10,3.03E-05,-0.559086568,0.167,0.525,0.746808396
DUSP2,3.06E-05,-0.819358873,0.076,0.373,0.754368594
DUOXA1,3.25E-05,-0.506886556,0.121,0.441,0.803092781
GSE1,3.27E-05,-0.381064098,0.015,0.271,0.806294183
EBF4,3.35E-05,-0.32448057,0.015,0.271,0.827847833
BCAM,3.49E-05,-0.798552502,0.652,0.932,0.860564615
PIM3,3.91E-05,-0.614263275,0.379,0.695,0.965601202
MFHAS1,3.99E-05,-0.519798591,0.076,0.373,0.985586763
TUBB,4.16E-05,0.475678851,0.545,0.186,1
ITGA2,4.27E-05,0.781665402,0.742,0.424,1
MAOA,4.46E-05,-0.586636355,0.106,0.407,1
PXDC1,4.64E-05,-0.398191266,0.076,0.39,1
NEDD9,4.76E-05,-0.636289581,0.348,0.644,1
BAG3,4.78E-05,-0.553176012,0.136,0.458,1
EGR1,4.95E-05,-0.862190788,0.788,0.932,1
PPIC,5.05E-05,0.400843618,0.424,0.102,1
PPL,5.07E-05,-0.516181805,0.182,0.508,1
LBH,5.35E-05,-0.516181805,0.136,0.475,1
IFNGR1,5.73E-05,-0.551697588,0.258,0.593,1
CAV1,6.09E-05,-1.16175107,0.106,0.39,1
RASSF7,6.13E-05,-0.287281952,0,0.22,1
MPRIP,6.21E-05,-0.396216324,0.03,0.288,1
CEMIP2,6.23E-05,-0.689380396,0.152,0.458,1
LRG1,6.32E-05,-0.615782701,0.106,0.407,1
NTM,6.32E-05,-0.775568433,0,0.22,1
PGK1,6.41E-05,0.513976895,0.576,0.22,1
IFI27,6.46E-05,0.751537297,0.273,0.017,1
PRRC2A,6.51E-05,-0.341074769,0.03,0.288,1
JAM3,6.86E-05,-0.36244842,0.015,0.254,1
GATA6,6.86E-05,-0.36244842,0.015,0.254,1
TXNIP,6.99E-05,-0.621182689,0.258,0.627,1
SLC34A2,7.09E-05,-0.992623799,0.409,0.695,1
PGF,7.16E-05,0.531271177,0.273,0.017,1
JDP2,7.17E-05,-0.424785476,0.061,0.339,1
PSMB9,7.23E-05,0.438641471,0.333,0.051,1
C12orf49,7.53E-05,-0.390020058,0.061,0.339,1
CLDN4,7.57E-05,-0.794331591,0.53,0.831,1
PRDM2,7.59E-05,-0.3668655,0.076,0.373,1
DSTN,7.79E-05,-0.532845222,0.773,0.915,1
TXN,8.67E-05,0.857614255,0.833,0.593,1
PTP4A2,9.68E-05,-0.433555685,0.197,0.542,1
STARD7,9.83E-05,-0.42782593,0.182,0.508,1
FGFR2,0.000102099,-0.553176012,0.152,0.458,1
FNBP1L,0.000102962,-0.364243934,0.091,0.39,1
RHOB,0.000103691,-0.633819514,0.576,0.814,1
C4orf3,0.000109959,0.474285615,0.561,0.237,1
RHOBTB2,0.000111038,-0.375154708,0.045,0.305,1

FAM189A2,0.000111038,-0.338628832,0.045,0.305,1
H2AFZ,0.000116125,0.417111231,0.606,0.237,1
TSC22D1,0.0001172,-0.713736248,0.879,0.966,1
PHYHD1,0.000117514,-0.468412408,0.136,0.441,1
NPNT,0.000119064,-0.533719847,0.03,0.271,1
CDC14B,0.000125818,-0.378068977,0.03,0.271,1
CEMIP,0.000130283,0.531271177,0.258,0.017,1
PLXNA2,0.00014038,-0.463779607,0.106,0.39,1
SOCS2,0.000140795,-0.566592802,0.061,0.322,1
VPS37B,0.000145405,-0.430937703,0.167,0.475,1
NEO1,0.000147265,-0.285486438,0.015,0.237,1
S100A2,0.000164579,1.204316454,0.97,0.847,1
ALDH3A1,0.000173679,-0.932725556,0.364,0.661,1
HMGN3,0.000178528,-0.518236387,0.652,0.831,1
TYMP,0.000186894,0.361810886,0.348,0.068,1
PKM,0.000187534,0.460444337,0.803,0.542,1
CXCL3,0.000189379,-0.746713571,0.182,0.458,1
SERPINB4,0.000194342,1.390234108,0.212,0,1
FARP1,0.000204374,-0.387043382,0.076,0.339,1
CHD3,0.00020646,-0.469179595,0.197,0.492,1
HLA-DPB1,0.00021321,0.70861365,0.348,0.085,1
TPI1,0.000219671,0.537589452,0.773,0.559,1
ZFAND5,0.000222032,-0.625318463,0.379,0.644,1
LAMA5,0.000227324,-0.421813909,0.197,0.508,1
RYBP,0.000228208,-0.315784699,0.061,0.322,1
OTULIN,0.000228691,-0.320013154,0.045,0.288,1
SLCO3A1,0.000239967,-0.543180177,0.152,0.424,1
FRMD4B,0.000241756,-0.378562459,0.106,0.39,1
MFSD10,0.000245534,-0.430567828,0.258,0.559,1
MRPS17,0.000247017,0.384530963,0.333,0.068,1
HIST1H1E,0.000248166,-0.451257687,0.091,0.356,1
LINC-PINT,0.000256606,-0.468412408,0.136,0.424,1
OAT,0.000256776,-0.483679165,0.485,0.729,1
TACC2,0.000259506,-0.463779607,0.106,0.373,1
MAP3K11,0.000261083,-0.287281952,0,0.186,1
SNRNP27,0.000265616,-0.341074769,0.03,0.254,1
MAP2K3,0.00026617,-0.401217005,0.091,0.356,1
POLR2L,0.000268878,-0.466144506,0.727,0.898,1
ZSWIM6,0.000272628,-0.322215742,0.03,0.254,1
SUPT5H,0.000272628,-0.359690448,0.03,0.254,1
KREMEN1,0.000272628,-0.322215742,0.03,0.254,1
OAZ2,0.000279526,-0.303106919,0.03,0.254,1
SGMS2,0.000281815,-0.511085322,0.106,0.373,1
IL6R,0.00028557,-0.390020058,0.061,0.305,1
MALSU1,0.000286297,-0.283741594,0.03,0.254,1
UBC,0.000292891,-0.486791777,0.985,1,1
RND1,0.00029659,-0.32448057,0.015,0.22,1
PRSS12,0.000300131,-0.305115245,0.015,0.22,1
TLCD2,0.000300131,-0.285486438,0.015,0.22,1
EFEMP1,0.000308123,-0.541599141,0.242,0.525,1

ALDH1A1,0.000312247,-0.451257687,0.394,0.746,1
CCND3,0.000331376,-0.336248801,0.061,0.305,1
CHST12,0.000331376,-0.336248801,0.061,0.305,1
ITPKC,0.000331392,-0.390020058,0.061,0.305,1
PRSS23,0.000335556,0.876929398,0.561,0.254,1
SAT1,0.000337897,0.856126421,1,0.983,1
MIF,0.000340356,0.550966978,0.924,0.797,1
ANKRD65,0.000347413,-0.317870272,0.061,0.305,1
ANXA3,0.000352936,-0.387043382,0.061,0.305,1
KRR1,0.00036103,-0.3952412,0.121,0.39,1
ANK3,0.000381359,-0.490699593,0.227,0.508,1
THBS1,0.0003923,-0.576788569,0.152,0.458,1
ATP5F1D,0.00040499,0.358027262,0.682,0.356,1
ID1,0.000413466,-0.828081122,0.833,0.966,1
VEZF1,0.000413584,-0.40866581,0.136,0.407,1
G3BP2,0.000414064,-0.357007361,0.045,0.271,1
GOLGA8B,0.000418544,-0.325249802,0.091,0.356,1
PIGR,0.00041902,0.850221572,0.227,0.017,1
TGM2,0.000429544,0.452958774,0.273,0.034,1
MRPS6,0.000431224,0.540167577,0.515,0.237,1
PSIP1,0.00043506,-0.412559173,0.167,0.458,1
ZNF711,0.00044713,-0.320013154,0.045,0.271,1
PLXNA1,0.000447153,-0.338628832,0.045,0.271,1
KITLG,0.000454934,-0.421618197,0.136,0.407,1
TXLNA,0.000463907,-0.301154127,0.045,0.271,1
TUBGCP2,0.000463907,-0.301154127,0.045,0.271,1
DDB1,0.000467426,-0.344615127,0.121,0.39,1
NR1D1,0.000467426,-0.344615127,0.121,0.39,1
SHB,0.000472605,-0.430937703,0.182,0.458,1
C1orf21,0.000474584,-0.278790491,0.061,0.305,1
MAL2,0.000477815,-0.378981786,0.182,0.475,1
KDM6B,0.000480069,-0.392363998,0.121,0.39,1
H1F0,0.000485011,-0.524321149,0.152,0.424,1
NOP53,0.000486829,-0.470382342,0.515,0.746,1
ZBTB21,0.000488095,-0.315784699,0.076,0.322,1
PLD3,0.000501746,-0.405676653,0.136,0.407,1
SERPINF1,0.000503227,-0.632931647,0.864,0.932,1
EBPL,0.00050493,-0.465143213,0.333,0.61,1
HLA-DPA1,0.000506255,0.575214524,0.348,0.085,1
APEX1,0.000511116,0.323675757,0.47,0.169,1
CTDSPL,0.000516152,-0.327337136,0.106,0.373,1
AK2,0.000516542,0.339443073,0.348,0.085,1
SLC16A12,0.000528589,-0.26710407,0,0.169,1
TUBA1A,0.000539253,-0.359690448,0.03,0.237,1
LINC01578,0.000542349,-0.434769564,0.333,0.644,1
INSR,0.000551423,-0.449553381,0.152,0.424,1
SPTSSA,0.000554001,-0.49382712,0.258,0.525,1
GJB3,0.000567966,-0.283741594,0.03,0.237,1
SH3RF1,0.000567966,-0.283741594,0.03,0.237,1
CENPX,0.000567966,-0.283741594,0.03,0.237,1

PBXIP1,0.000582105,-0.264112788,0.03,0.237,1
TEAD3,0.000582105,-0.264112788,0.03,0.237,1
LCA5,0.000582105,-0.264112788,0.03,0.237,1
SVOPL,0.000582105,-0.264112788,0.03,0.237,1
HACD1,0.000582105,-0.264112788,0.03,0.237,1
BCL6,0.000593145,-0.378981786,0.167,0.458,1
ADRB1,0.000595536,-0.305115245,0.015,0.203,1
NFIB,0.000632573,-0.446728029,0.273,0.559,1
SF3B2,0.000642864,-0.364243934,0.091,0.339,1
HIST1H1C,0.000677964,-0.591143862,0.121,0.373,1
FABP5,0.000681631,0.398963884,0.424,0.153,1
TRIM16,0.000693246,-0.282045304,0.03,0.237,1
ADD1,0.000693871,-0.299254594,0.061,0.288,1
ATN1,0.000693871,-0.299254594,0.061,0.288,1
CDH1,0.000703733,-0.612857824,0.182,0.441,1
S100A14,0.000709274,-0.665517488,0.576,0.797,1
RIC3,0.000716737,-0.421394887,0.076,0.305,1
CFL2,0.000726643,-0.280395566,0.061,0.288,1
ELMSAN1,0.000728516,-0.346965942,0.091,0.339,1
ADIRF,0.000729949,-0.628532144,0.636,0.847,1
CELF2,0.000732511,-0.462617549,0.045,0.254,1
RARRES2,0.000743787,-0.264112788,0.015,0.203,1
ARL4A,0.000760105,0.430590961,0.348,0.102,1
KPNA4,0.000793039,-0.333932045,0.061,0.288,1
CDC42BPA,0.000797781,-0.331676071,0.061,0.288,1
NKX2-1,0.000811554,-0.466605652,0.242,0.508,1
ARRDC3,0.000821366,0.567287608,0.424,0.169,1
YTHDF2,0.00083396,-0.378562459,0.121,0.373,1
EGFR,0.000844647,-0.540262693,0.636,0.814,1
CP,0.000877424,0.949280242,0.636,0.39,1
OSBPL9,0.000893269,-0.301154127,0.045,0.254,1
DENND2C,0.000893269,-0.301154127,0.045,0.254,1
AC016831.5,0.000893269,-0.301154127,0.045,0.254,1
LDHA,0.000906613,0.557228779,0.758,0.627,1
LOX,0.00091159,0.316902529,0.242,0.034,1
FAF1,0.000947291,-0.374054674,0.212,0.492,1
CCDC57,0.000960926,-0.262679979,0.045,0.254,1
NDFIP2,0.00096203,-0.401421483,0.242,0.508,1
OXR1,0.001014951,-0.278790491,0.076,0.305,1
LPIN2,0.001022341,-0.359690448,0.03,0.22,1
ANKRD12,0.001042042,-0.483679165,0.348,0.61,1
AC068587.4,0.001062468,-0.26710407,0,0.153,1
ABI2,0.001063041,-0.292147707,0.106,0.356,1
COLCA1,0.00106871,-0.398191266,0.106,0.339,1
LGALS7,0.001080879,-0.303106919,0.03,0.22,1
FGGY,0.001110239,-0.283741594,0.03,0.22,1
ISYNA1,0.001113015,-0.315556406,0.197,0.475,1
KMT2E,0.001132896,-0.384143491,0.167,0.424,1
NYNRIN,0.001139407,-0.264112788,0.03,0.22,1
AKR1C2,0.001146585,-0.727348246,0.061,0.271,1

SFSWAP,0.001157929,-0.261286744,0.045,0.254,1
DUSP14,0.001165445,-0.256078453,0.091,0.339,1
PLCG2,0.001180285,-1.608864035,0.485,0.627,1
CASZ1,0.001182653,-0.32448057,0.015,0.186,1
CASP4,0.001206163,0.361810886,0.318,0.085,1
CPM,0.001233488,-0.265586881,0.015,0.186,1
TIAM1,0.001239204,-0.317870272,0.061,0.271,1
LAYN,0.001251736,-0.295606817,0.091,0.322,1
KLK11,0.00125314,-0.592385424,0.303,0.576,1
CSTB,0.001273818,0.762960803,0.758,0.593,1
KRT7,0.001280399,-0.724171002,0.485,0.763,1
ITGB8,0.001285875,-0.278564735,0.182,0.458,1
SLC44A2,0.001299979,-0.299254594,0.061,0.271,1
IER3,0.001356277,-0.483679165,0.818,0.932,1
ERBB3,0.00136241,-0.280395566,0.061,0.271,1
SERPINB3,0.001380731,0.441177977,0.288,0.068,1
S100A13,0.00139289,-0.310273595,0.182,0.475,1
EIF2S2,0.001400671,0.347262577,0.545,0.271,1
ADNP,0.001402091,-0.311776514,0.091,0.322,1
SON,0.001404197,-0.40275917,0.515,0.763,1
PSMB8,0.001420776,0.336702437,0.288,0.068,1
VMO1,0.001421168,0.625519606,0.258,0.051,1
NGFR,0.00142426,-0.418908909,0.197,0.458,1
CD302,0.001475222,-0.387043382,0.076,0.288,1
MT-ND4L,0.001495749,-0.583984071,0.621,0.797,1
NIPBL,0.001497592,-0.293854606,0.091,0.322,1
IL6ST,0.00153882,-0.504352733,0.394,0.627,1
SPAG9,0.001597817,-0.275707259,0.091,0.322,1
ETS2,0.001619611,-0.483679165,0.742,0.831,1
DOCK5,0.001658353,-0.571839353,0.091,0.305,1
SSB,0.001660881,0.423211431,0.5,0.254,1
IER2,0.001681074,-0.676324243,0.636,0.797,1
KCNN4,0.001692845,-0.301154127,0.045,0.237,1
BAIAP2,0.001718438,-0.350196159,0.273,0.542,1
IGF1R,0.001719378,-0.327337136,0.121,0.356,1
ZNF395,0.001753591,-0.297406169,0.076,0.288,1
PDE7A,0.001758035,-0.282045304,0.045,0.237,1
SNHG19,0.001760571,-0.373255175,0.136,0.373,1
PGRMC1,0.001792195,-0.339289256,0.242,0.508,1
ATXN7,0.001824156,-0.301154127,0.045,0.237,1
CUX1,0.001824156,-0.262679979,0.045,0.237,1
BCL9L,0.001824156,-0.262679979,0.045,0.237,1
WBP11,0.001824156,-0.262679979,0.045,0.237,1
PDLIM1,0.001828362,0.497212012,0.818,0.559,1
SEPTIN9,0.001838318,-0.361059878,0.197,0.458,1
ATP5MC3,0.001849476,0.381692161,0.727,0.475,1
UBE3A,0.001863112,-0.292147707,0.121,0.356,1
RDX,0.001874027,-0.464820138,0.152,0.373,1
STARD10,0.00189561,-0.281332686,0.182,0.458,1
PSMA2,0.001903671,0.312180118,0.47,0.203,1

HSPA4L,0.001914766,-0.268666274,0.121,0.373,1
SERTAD2,0.001958921,-0.331676071,0.091,0.305,1
TFPI2,0.001968525,-0.703644849,0.03,0.203,1
PLEC,0.001980406,-0.307971811,0.136,0.373,1
CLDN10,0.001991811,0.337350694,0.379,0.136,1
MDK,0.002127655,0.48210512,0.5,0.237,1
GAPDH,0.002130953,0.692909567,0.955,0.831,1
WDR45B,0.002156553,-0.313754163,0.197,0.458,1
AHI1,0.002161355,-0.290484384,0.136,0.373,1
TNKS1BP1,0.002188173,-0.277228287,0.076,0.288,1
ANKUB1,0.002198864,-0.264112788,0.03,0.203,1
FKBP5,0.002198864,-0.264112788,0.03,0.203,1
PSTPIP2,0.002198864,-0.264112788,0.03,0.203,1
GALNT2,0.00219898,-0.283741594,0.03,0.203,1
AKAP9,0.002237357,-0.31672573,0.333,0.61,1
CAV2,0.002250093,-0.50367064,0.258,0.492,1
MDM4,0.002260852,-0.437385513,0.136,0.373,1
EFNA1,0.002268606,-0.386311328,0.333,0.593,1
AL355075.4,0.002313467,-0.258612609,0.076,0.288,1
CCND1,0.002317714,-0.417765048,0.379,0.627,1
GADD45B,0.00233194,-0.630426533,0.348,0.559,1
HS6ST1,0.002349042,-0.364243934,0.106,0.322,1
THBD,0.002396603,-0.265586881,0.015,0.169,1
RND3,0.002472096,0.577597175,0.742,0.542,1
F11R,0.002495245,-0.448632218,0.424,0.678,1
REL,0.002510134,-0.280395566,0.061,0.254,1
GPS2,0.002510134,-0.280395566,0.061,0.254,1
ARL4C,0.002607773,-0.468412408,0.136,0.356,1
MT1E,0.002624421,-0.814556216,0.682,0.797,1
APOL1,0.002625643,0.340749271,0.182,0.017,1
SCPEP1,0.002628538,-0.504143267,0.455,0.678,1
UXS1,0.002629348,-0.261286744,0.061,0.254,1
DDIT3,0.002629348,-0.261286744,0.061,0.254,1
PER1,0.002629348,-0.261286744,0.061,0.254,1
LENG8,0.002629348,-0.261286744,0.061,0.254,1
ARGLU1,0.00263096,-0.440052232,0.364,0.61,1
DNAJB1,0.002689899,-0.507746925,0.364,0.61,1
TMED4,0.002699008,-0.287281952,0.167,0.407,1
NDUFAF8,0.002710638,-0.274225799,0.091,0.305,1
STOML2,0.002773549,0.326748591,0.364,0.136,1
PSMC6,0.002795745,-0.342323316,0.136,0.356,1
LMO3,0.002811281,-0.384143491,0.136,0.373,1
TNFSF10,0.002882976,0.759774872,0.5,0.271,1
SNRPG,0.002904517,0.389891358,0.47,0.237,1
TBCA,0.002919927,0.347262577,0.515,0.237,1
ZBTB38,0.002924894,-0.278790491,0.061,0.254,1
ABI1,0.002971915,-0.315784699,0.076,0.271,1
ZFHX3,0.002971915,-0.315784699,0.076,0.271,1
DNAJB6,0.00301086,-0.424785476,0.318,0.542,1
TMEM45A,0.003025244,0.522221437,0.182,0.017,1

PPP4R1,0.00304819,-0.340088311,0.152,0.373,1
MED21,0.003066533,-0.259931464,0.061,0.254,1
NDUFB3,0.003069843,0.297680549,0.273,0.068,1
RPS11,0.003074111,-0.461952977,0.955,0.966,1
REV1,0.00310147,-0.323214493,0.121,0.339,1
RHOBTB3,0.003116787,-0.483679165,0.197,0.424,1
SDR16C5,0.003145309,-0.297406169,0.076,0.271,1
EPAS1,0.003156151,-0.583214838,0.485,0.644,1
EDN1,0.003159128,-0.621182689,0.152,0.373,1
AUTS2,0.003191212,-0.292147707,0.121,0.339,1
COX5B,0.003216709,0.366627902,0.773,0.475,1
TSTD1,0.003292978,-0.41451714,0.394,0.593,1
FOXC1,0.003308086,-0.359197134,0.121,0.339,1
EIF5A,0.003366081,0.405289523,0.53,0.305,1
ADGRF5,0.003383125,-0.401217005,0.061,0.254,1
PRKCE,0.003408034,-0.262679979,0.045,0.22,1
GGNBP2,0.003433104,-0.331676071,0.091,0.288,1
KRT23,0.003445056,0.313157885,0.136,0,1
AGR3,0.003447534,0.398549376,0.136,0,1
HNRNPH3,0.003449065,-0.347617615,0.152,0.39,1
MMP10,0.003450758,1,0.136,0,1
SIPA1L1,0.003451634,-0.274225799,0.121,0.339,1
SLC25A44,0.003535301,-0.282045304,0.045,0.22,1
VEGFA,0.003581154,-0.427095637,0.333,0.576,1
TPBG,0.00363463,-0.328109456,0.318,0.559,1
CAVIN1,0.003663018,-0.307971811,0.121,0.339,1
STIM2,0.003666268,-0.331676071,0.091,0.288,1
ZC3H12A,0.003675643,-0.368201947,0.167,0.39,1
GNL1,0.003687552,-0.306140979,0.152,0.373,1
NT5DC1,0.003702539,-0.290484384,0.121,0.339,1
C1QBP,0.003708799,0.387587521,0.591,0.373,1
CITED2,0.00374543,-0.516181805,0.152,0.356,1
LSM3,0.003812922,-0.319292347,0.182,0.407,1
CXCL8,0.00387074,-0.615756799,0.576,0.763,1
NT5DC2,0.003893714,-0.346965942,0.091,0.288,1
BACE2,0.003924115,0.297680549,0.409,0.169,1
KLF6,0.003985708,-0.623786049,0.545,0.746,1
SH2D3A,0.004006616,-0.272782382,0.121,0.339,1
AP003498.2,0.004028848,-0.261286744,0.045,0.22,1
CDK2AP1,0.004062136,-0.356567247,0.182,0.424,1
SRGAP3,0.004085278,-0.366109569,0.288,0.542,1
PPHLN1,0.004165962,-0.277228287,0.091,0.288,1
COL7A1,0.004168053,-0.299254594,0.167,0.407,1
VAMP2,0.004220218,-0.344615127,0.121,0.322,1
PHACTR3,0.004224185,-0.384143491,0,0.119,1
CDC42,0.004294048,-0.334815779,0.364,0.61,1
HDAC5,0.004301551,-0.264112788,0.03,0.186,1
RASD1,0.0043988,-0.40275917,0.227,0.475,1
AHR,0.004431306,-0.414293102,0.515,0.729,1
TMEM98,0.004434595,-0.277228287,0.091,0.288,1

P4HA1,0.004449433,0.270359943,0.258,0.068,1
TLE5,0.004532889,-0.391618612,0.561,0.712,1
LMO7,0.004543024,-0.40275917,0.53,0.712,1
GSTO1,0.004561741,0.304566934,0.455,0.203,1
SEM1,0.00456669,0.306801939,0.636,0.373,1
NCOR2,0.004578138,-0.378562459,0.121,0.322,1
SRSF10,0.004591564,-0.369346489,0.242,0.475,1
NDUFB2,0.004627273,0.320849713,0.439,0.203,1
COA4,0.004638686,0.253286429,0.258,0.068,1
ADI1,0.004669259,-0.287281952,0.152,0.373,1
RIOK3,0.004737589,-0.300913818,0.167,0.39,1
TXNDC5,0.004763646,-0.280395566,0.061,0.237,1
SYNE1,0.004763646,-0.299254594,0.061,0.237,1
DDX5,0.004777546,-0.485046001,0.879,0.966,1
RGMB,0.004785824,-0.275707259,0.091,0.288,1
MAP1LC3B,0.004785824,-0.275707259,0.091,0.288,1
SGK1,0.004818924,-0.560300446,0.227,0.458,1
LIMCH1,0.004949587,-0.297406169,0.061,0.237,1
SELENBP1,0.004956397,-0.309849709,0.121,0.322,1
TRIM8,0.004957619,-0.349823418,0.197,0.407,1
SLC16A1,0.004986426,0.265063597,0.197,0.034,1
CIR1,0.004986643,-0.253673559,0.136,0.356,1
PRSS8,0.004997612,-0.275707259,0.106,0.305,1
SLC66A2,0.004997612,-0.275707259,0.106,0.305,1
DDX24,0.00502931,-0.441858989,0.273,0.475,1
FOSL2,0.005119199,-0.270003961,0.152,0.373,1
GSTA1,0.005189469,-0.4675595,0.091,0.271,1
HMGA1,0.005228791,0.303211655,0.394,0.169,1
HLA-DMA,0.005244351,0.264513685,0.258,0.068,1
CD44,0.005417939,0.365878256,0.439,0.203,1
PSMA3,0.005433795,0.308878755,0.394,0.169,1
SUSD6,0.005443765,-0.384143491,0.182,0.39,1
CREBZF,0.005444121,-0.259931464,0.061,0.237,1
C11orf96,0.005508978,-0.320013154,0.015,0.153,1
TMEM87A,0.005564953,-0.349823418,0.197,0.407,1
PURB,0.005712929,-0.274225799,0.106,0.305,1
ALCAM,0.005713218,-0.589844722,0.394,0.61,1
DDX17,0.005731373,-0.362798589,0.379,0.661,1
MET,0.005757669,-0.407328279,0.409,0.627,1
KLF4,0.005888586,0.352822103,0.485,0.22,1
HSPG2,0.006001667,-0.306140979,0.152,0.356,1
PTPN13,0.006006593,-0.307971811,0.136,0.339,1
HSBP1,0.006017529,0.351061645,0.576,0.373,1
AATK,0.006038383,-0.282045304,0.045,0.203,1
ATP2C2,0.006106544,0.281192426,0.273,0.085,1
CAPN8,0.006127483,-0.256078453,0.106,0.305,1
TSPO,0.006138267,0.37788744,0.682,0.441,1
B4GALT1,0.006249452,-0.292995603,0.242,0.475,1
TPM1,0.006387812,-0.412074424,0.348,0.559,1
VDAC2,0.006446397,0.293252837,0.667,0.424,1

BCL3,0.006577456,-0.306140979,0.152,0.356,1
DYNLL2,0.006596864,-0.272782382,0.121,0.322,1
GRINA,0.006723173,-0.295606817,0.091,0.271,1
XPC,0.006724717,-0.331676071,0.091,0.271,1
NME2,0.006750923,0.35379003,0.924,0.746,1
6-Mar,0.006784322,-0.263630684,0.197,0.424,1
MUC1,0.006854655,-0.606232347,0.227,0.458,1
RPL27A,0.006857754,-0.489496491,0.939,0.966,1
SLC20A1,0.006866056,-0.421986842,0.212,0.441,1
TC2N,0.007122326,-0.254860474,0.121,0.322,1
CFH,0.007144146,-0.426804643,0.561,0.746,1
RAB34,0.007149261,-0.313754163,0.212,0.424,1
FOXO3,0.007197844,-0.271375561,0.152,0.356,1
SEC31A,0.007326645,-0.288862988,0.121,0.322,1
ASL,0.00734228,-0.306140979,0.136,0.339,1
CD46,0.007583186,-0.435316143,0.318,0.525,1
GTF2I,0.00758472,-0.276083745,0.348,0.593,1
HRAS,0.007626373,-0.263630684,0.212,0.441,1
TALDO1,0.007710916,0.320641697,0.333,0.136,1
TRA2B,0.007737068,-0.415037499,0.379,0.627,1
BIRC3,0.007775859,-0.404111908,0.167,0.373,1
SMARCA1,0.007866974,-0.253673559,0.152,0.356,1
MRNIP,0.008003568,-0.311776514,0.076,0.254,1
XRRA1,0.00809257,-0.264112788,0.03,0.169,1
LY6E,0.008174213,0.354169786,0.742,0.559,1
ABCC3,0.008198805,-0.384143491,0.167,0.356,1
KRT5,0.008393585,0.575214524,0.788,0.576,1
DDX6,0.00839681,-0.275707259,0.106,0.288,1
ANKRD11,0.008400754,-0.303770075,0.242,0.458,1
LAP3,0.008456566,0.259092051,0.212,0.051,1
FBLN1,0.00854883,-0.464820138,0.121,0.305,1
NDUFB7,0.008549281,0.271902107,0.485,0.254,1
HCAR2,0.008624863,-0.551697588,0.258,0.441,1
AHNAK,0.00866014,-0.543313865,0.394,0.61,1
RBM22,0.008992927,-0.275707259,0.106,0.288,1
PDIA3,0.009034883,0.455632908,0.545,0.356,1
RBMS3,0.009093672,-0.310273595,0.197,0.407,1
H2AFY,0.009137989,0.268883284,0.318,0.119,1
APOE,0.009192092,-0.435511882,0.015,0.136,1
PLIN2,0.009518109,0.281192426,0.212,0.051,1
COX7B,0.009570175,0.417542681,0.545,0.322,1
PLAAT4,0.009572931,0.451225807,0.545,0.339,1
SERF2,0.009659632,0.385243581,0.909,0.78,1
UQCC2,0.009756879,0.263554765,0.394,0.186,1
NINJ1,0.009845693,-0.259931464,0.061,0.22,1
HERPUD1,0.010016672,-0.288663182,0.242,0.475,1
7-Mar,0.010146658,-0.297406169,0.076,0.237,1
LARP1B,0.010146658,-0.297406169,0.076,0.237,1
FCGRT,0.010241362,-0.294506279,0.212,0.424,1
RPS17,0.01034569,-0.455110013,0.924,0.915,1

RPL13A,0.010386116,-0.585864348,0.985,0.983,1
NFKBIZ,0.010423627,-0.306140979,0.394,0.627,1
COX7A2,0.010429927,0.329235283,0.818,0.661,1
HEXIM1,0.010670828,-0.272782382,0.121,0.305,1
SBNO2,0.010688796,0.269588242,0.303,0.119,1
FGFBP1,0.01069316,0.828053163,0.318,0.136,1
PSME4,0.010695229,-0.256078453,0.106,0.288,1
MIA3,0.010705556,-0.259931464,0.076,0.237,1
KDM5B,0.010950326,-0.329873829,0.288,0.492,1
SCNN1A,0.01107602,-0.520544459,0.273,0.441,1
MGST1,0.011241207,-0.363993792,0.818,0.932,1
ECI2,0.011288334,-0.259931464,0.076,0.237,1
COX5A,0.011290946,0.273464311,0.394,0.186,1
ARID1B,0.011373864,-0.311993706,0.197,0.39,1
USP34,0.011445579,-0.281332686,0.182,0.39,1
DAP,0.01145749,-0.254860474,0.136,0.322,1
LRRFIP1,0.011574628,-0.324809352,0.576,0.746,1
ASAH1,0.011600089,-0.363384931,0.197,0.39,1
NOLC1,0.011785465,-0.271375561,0.136,0.322,1
CCDC186,0.012128986,-0.319292347,0.167,0.356,1
S100A10,0.012147735,0.564430848,0.758,0.763,1
EIF3L,0.01223241,-0.296052162,0.242,0.441,1
PNRC1,0.012304211,-0.326137888,0.515,0.746,1
COX6A1,0.012424777,0.271664959,0.848,0.678,1
GDI2,0.012453134,0.295721696,0.318,0.136,1
MYL6,0.012471574,0.506011736,0.879,0.746,1
PRKAR1A,0.012471762,-0.427484625,0.303,0.475,1
IGFBP7,0.012472876,-0.643022268,0.621,0.712,1
SRSF6,0.012501037,-0.263630684,0.212,0.424,1
SPTBN1,0.01254184,-0.306140979,0.136,0.322,1
BTG2,0.01262675,-0.610735901,0.455,0.661,1
PTGES3,0.012628743,0.328574557,0.561,0.373,1
SPPL2A,0.012837172,-0.295606817,0.091,0.254,1
ZNF503,0.012884435,-0.343357876,0.212,0.407,1
EIF5,0.012955388,-0.31672573,0.333,0.576,1
ARIH1,0.013071808,-0.253673559,0.106,0.288,1
TRAM1,0.013096593,-0.542572854,0.333,0.508,1
STAT2,0.013154815,0.253286429,0.227,0.068,1
SAA1,0.013212707,0.582689645,0.303,0.119,1
ID4,0.013688061,-0.386311328,0.318,0.508,1
SYTL1,0.013698703,-0.287281952,0.303,0.508,1
NONO,0.01373908,0.277780991,0.409,0.203,1
IRX3,0.013791023,-0.350512713,0.348,0.559,1
SIPA1L2,0.013852259,-0.293854606,0.106,0.271,1
PLXNB2,0.014066961,-0.378981786,0.197,0.373,1
GPRC5A,0.014186591,-0.539271491,0.848,0.881,1
TXNDC17,0.014299373,0.428728436,0.591,0.373,1
TNIP2,0.014315695,-0.292147707,0.121,0.288,1
ADAM28,0.014685167,0.610296983,0.258,0.102,1
DESI2,0.014812577,-0.25732873,0.106,0.271,1

HIST1H1D,0.015037817,-0.303106919,0.03,0.153,1
PFDN2,0.01529402,0.283033773,0.409,0.22,1
KLK10,0.01535133,-0.818863356,0.106,0.271,1
IGFBP6,0.015396469,0.732539489,0.485,0.271,1
HIVEP3,0.015444513,-0.264112788,0.03,0.153,1
EPPK1,0.015698296,-0.25732873,0.091,0.254,1
RPL7,0.015751847,-0.401578085,0.955,0.949,1
HS3ST1,0.015811873,0.511084187,0.318,0.153,1
COBLL1,0.015826699,-0.25732873,0.106,0.271,1
PER2,0.015966016,-0.299254594,0.303,0.508,1
JUND,0.016050496,-0.255819394,0.879,0.983,1
DLL1,0.016134212,-0.409678583,0.182,0.356,1
DDI2,0.016267,-0.264112788,0.03,0.153,1
PLXNB1,0.016348599,-0.256078453,0.106,0.271,1
TMSB10,0.016391287,0.646852398,0.803,0.678,1
TSPAN8,0.01649198,0.385736725,0.258,0.102,1
RBM27,0.016772403,-0.272782382,0.136,0.305,1
CLDN3,0.016874133,-0.267361258,0.091,0.254,1
SERPINB13,0.016908346,0.601209733,0.258,0.102,1
IL1RAP,0.017031375,0.323675757,0.333,0.153,1
GSN,0.017086413,0.416422265,0.318,0.136,1
SNHG14,0.017357032,-0.31740194,0.182,0.356,1
S100P,0.017600781,0.61268923,0.348,0.169,1
EIF3E,0.017664045,-0.316079216,0.636,0.78,1
PKP1,0.017711929,-0.297406169,0.061,0.203,1
STAT3,0.017880043,-0.427095637,0.455,0.61,1
RPS20,0.017980089,-0.460959088,0.924,0.966,1
SMARCC1,0.018153666,-0.254860474,0.136,0.305,1
RBBP6,0.018217401,-0.361688641,0.121,0.271,1
TIPARP,0.018273432,-0.291518945,0.227,0.407,1
IRF6,0.018358799,-0.278564735,0.227,0.424,1
LAMTOR5,0.018651443,0.282348895,0.591,0.39,1
LITAF,0.01881847,-0.328109456,0.273,0.458,1
EID1,0.018895798,-0.43637345,0.667,0.729,1
BTG1,0.019028171,-0.306699406,0.485,0.729,1
DSP,0.019075894,-0.422617639,0.455,0.61,1
CRIP2,0.019482339,-0.253673559,0.152,0.322,1
KRT4,0.019486957,-0.530395664,0.121,0.288,1
PGC,0.019718892,-0.466605652,0.015,0.119,1
TXNDC11,0.020176038,-0.278790491,0.076,0.22,1
AASS,0.020584765,-0.32587045,0.227,0.407,1
SEC61G,0.02065262,0.275112792,0.561,0.373,1
ZC3HAV1,0.020817935,-0.25732873,0.076,0.22,1
PTMS,0.020871693,-0.299254594,0.606,0.763,1
RPS10,0.020936403,0.350058318,0.97,0.949,1
IER5,0.021159187,-0.270003961,0.152,0.322,1
RARRES1,0.021164765,0.588889461,0.182,0.051,1
TRIP10,0.0211809,-0.253673559,0.152,0.322,1
TNS1,0.021234455,-0.337322635,0.212,0.39,1
SAR1A,0.021246727,-0.258612609,0.091,0.237,1

AGR2,0.021284481,0.818796567,0.485,0.288,1
PTTG1IP,0.021295714,-0.267361258,0.182,0.356,1
PRSS22,0.021344635,-0.252516534,0.152,0.322,1
SVIL,0.021438956,-0.251388282,0.182,0.356,1
RPL3,0.021622829,-0.399567533,1,0.983,1
MED24,0.021806189,-0.262679979,0.045,0.169,1
RORA,0.021806912,-0.282045304,0.045,0.169,1
RABAC1,0.021922796,-0.281332686,0.212,0.39,1
SELENOM,0.021980552,-0.310273595,0.242,0.407,1
LAMB2,0.022513348,-0.251388282,0.167,0.339,1
WFDC2,0.02274104,1.17185883,0.894,0.797,1
SLC4A4,0.022831094,0.284313325,0.318,0.153,1
RPL31,0.022884463,-0.351323314,0.924,0.966,1
DDX3X,0.023064989,-0.316473665,0.333,0.525,1
CNPY2,0.023243639,-0.267361258,0.182,0.356,1
SOX9,0.024110423,-0.429446853,0.394,0.559,1
PRPF4B,0.024672511,-0.320448816,0.273,0.441,1
KRT17,0.024832588,-0.494940714,0.97,1,1
CRIP1,0.024858025,0.725239968,0.333,0.169,1
NME3,0.025152122,-0.352272976,0.288,0.441,1
SLC2A1,0.025404698,0.408997572,0.303,0.153,1
N4BP2L2,0.025519083,-0.312167913,0.303,0.508,1
SCNN1B,0.025590189,-0.272782382,0.106,0.254,1
BEX3,0.025666109,-0.377884501,0.364,0.525,1
GOLGB1,0.026221526,-0.510758699,0.364,0.525,1
EHF,0.026498315,-0.263630684,0.53,0.729,1
SNHG15,0.02660151,-0.282766471,0.212,0.373,1
CALR,0.026682514,0.395305434,0.5,0.373,1
SLC25A5,0.026683471,0.288561904,0.697,0.508,1
CRNDE,0.026827481,-0.307601936,0.364,0.525,1
SCGB3A2,0.026996982,-0.644833957,0.076,0.22,1
ANAPC16,0.02734078,-0.280803742,0.318,0.508,1
KCNQ1OT1,0.027701846,-0.618389474,0.258,0.424,1
MALAT1,0.02810038,-0.515165922,1,1,1
SLC25A24,0.028194224,-0.256078453,0.121,0.271,1
YTHDC1,0.028387159,-0.279932496,0.227,0.39,1
SERPINB1,0.028501753,0.784477674,0.364,0.203,1
PPP2R2A,0.028829236,0.301220906,0.409,0.237,1
COPE,0.028988483,0.293928414,0.348,0.186,1
LAPTM4A,0.029018301,-0.295429505,0.47,0.61,1
PHIP,0.029407,-0.346175641,0.273,0.441,1
SPOCK3,0.029640332,-0.270003961,0.152,0.305,1
GLUL,0.029886575,-0.348164194,0.439,0.593,1
SPEN,0.030414189,-0.285739787,0.167,0.322,1
STK17A,0.030565961,-0.250106944,0.379,0.559,1
SEMA4A,0.030878044,-0.374054674,0.167,0.339,1
SFPQ,0.031157099,-0.316261019,0.455,0.644,1
CX3CL1,0.031237291,-0.296052162,0.227,0.39,1
EIF3F,0.031350021,-0.276083745,0.439,0.576,1
SDC1,0.033177762,-0.354396148,0.455,0.627,1

TNFRSF12A,0.033414076,-0.38746385,0.545,0.678,1
CXCL6,0.033421132,-0.448055255,0.182,0.339,1
MAFF,0.033564345,-0.306948986,0.273,0.441,1
LINC01436,0.033603634,-0.315784699,0.076,0.203,1
TM9SF2,0.034080975,-0.268666274,0.152,0.305,1
RPL10A,0.034263319,-0.306699406,0.97,0.983,1
RPL37A,0.034939591,-0.303649038,1,1,1
CBR3,0.035912533,-0.36244842,0.015,0.102,1
FGFR3,0.036054084,-0.306948986,0.273,0.424,1
SPATS2L,0.036485245,0.270564421,0.5,0.322,1
PMEPA1,0.036500509,0.293928414,0.318,0.169,1
ARPC3,0.037325967,0.283388461,0.5,0.322,1
MED13L,0.037484091,-0.252516534,0.152,0.305,1
BZW1,0.038329822,-0.250106944,0.318,0.492,1
METTL7A,0.038541603,-0.421986842,0.242,0.407,1
NKTR,0.038757356,-0.345463023,0.121,0.271,1
XIST,0.03909678,-0.3996149,0.197,0.373,1
EEF1G,0.039448985,-0.324345575,0.985,0.915,1
ATP5F1B,0.039616804,0.265670154,0.545,0.39,1
TRIM2,0.040108954,0.273464311,0.288,0.136,1
SMCHD1,0.040469272,-0.25732873,0.106,0.237,1
MUC16,0.041328936,0.271208337,0.106,0.017,1
MT-CO3,0.041819993,0.303994701,1,1,1
NORAD,0.042752598,-0.267361258,0.197,0.339,1
ANXA4,0.043375092,-0.274645126,0.273,0.424,1
TLE4,0.043609144,-0.27098014,0.227,0.39,1
EZR,0.043719519,-0.313754163,0.545,0.746,1
CCN2,0.044039714,-0.318720416,0.167,0.322,1
EIF3A,0.04407664,0.31358694,0.5,0.339,1
TRIB1,0.044942213,-0.40053793,0.379,0.559,1
TFF3,0.045662822,0.375905716,0.136,0.034,1
KLHL24,0.045978098,-0.252516534,0.167,0.305,1
UQCR10,0.046621047,0.269406095,0.591,0.424,1
RBM25,0.047541727,-0.377479761,0.348,0.492,1
S100A9,0.04784968,0.326748591,0.136,0.034,1
HSPD1,0.047884421,0.315151685,0.606,0.441,1
MPZL2,0.048010248,0.303912502,0.788,0.729,1
NABP1,0.04852308,-0.361688641,0.106,0.237,1
GSTP1,0.048995598,0.390530732,0.985,0.898,1
EPS8,0.049049545,-0.288862988,0.136,0.271,1
ARF4,0.049325133,-0.250106944,0.394,0.559,1
RACK1,0.049543064,-0.359583838,0.985,0.966,1
IGBP1,0.049842581,-0.254860474,0.136,0.271,1
ING2,0.049976972,-0.252516534,0.167,0.305,1
RPL10,0.050395217,-0.358822213,0.97,1,1
ZNF292,0.05115338,-0.284235077,0.167,0.305,1
CLDN1,0.052727707,-0.341871839,0.848,0.932,1
RPS3,0.054356475,-0.299254594,0.985,1,1
NASP,0.054547897,-0.384143491,0.258,0.373,1
HSP90B1,0.05691185,0.272145457,0.712,0.61,1

KRT6A,0.057987318,0.483965462,0.152,0.051,1
MUC4,0.059568225,0.398963884,0.409,0.254,1
CSF1,0.061411732,-0.277228287,0.091,0.203,1
SREBF1,0.063299795,-0.293854606,0.091,0.203,1
PPDPF,0.064045859,0.43742529,0.636,0.492,1
FLNA,0.064231138,-0.292995603,0.258,0.39,1
CTSC,0.064534308,0.540167577,0.318,0.186,1
WAC,0.064645839,-0.307971811,0.136,0.254,1
LRATD1,0.064751655,-0.277228287,0.091,0.203,1
GAS5,0.065366106,-0.35767128,0.833,0.814,1
BRD2,0.065718939,-0.28331305,0.288,0.441,1
PLAU,0.066839566,-0.272782382,0.091,0.203,1
DEFB1,0.068103705,0.59401212,0.121,0.034,1
NUFIP2,0.069146349,-0.372518166,0.303,0.441,1
LRRC8A,0.070468037,-0.255873247,0.303,0.441,1
MBIP,0.071187237,-0.292995603,0.258,0.39,1
TOP1,0.07131542,-0.346668786,0.5,0.593,1
CLDN7,0.07170644,0.362515499,0.455,0.339,1
CD63,0.072568058,-0.263630684,0.848,0.915,1
SRRM2,0.073523504,-0.331676071,0.53,0.61,1
JMJD1C,0.075172029,-0.31740194,0.182,0.305,1
AKR1C3,0.078612873,-0.279932496,0.167,0.288,1
MT-CYB,0.08116829,-0.35370855,1,0.983,1
PNISR,0.082773634,-0.277228287,0.5,0.61,1
TMBIM6,0.083341169,-0.3148304,0.667,0.729,1
NAMPT,0.084463053,-0.485949339,0.5,0.593,1
ATP5MPL,0.086784659,0.275935168,0.409,0.288,1
SNHG6,0.087215205,-0.271723912,0.47,0.542,1
ANXA2,0.08955096,0.416015929,0.97,0.949,1
PRRC2C,0.090346453,-0.303770075,0.576,0.678,1
PALLD,0.091178723,-0.251018408,0.318,0.441,1
FAM107B,0.091415395,-0.29781262,0.333,0.458,1
TP53TG1,0.091635398,-0.282766471,0.212,0.322,1
PSME1,0.095410559,0.253286429,0.606,0.492,1
IRX2,0.096229033,-0.278395989,0.364,0.475,1
INHBB,0.096357151,-0.315784699,0.076,0.169,1
NPM1,0.098767468,0.280039157,0.97,0.949,1
ERO1A,0.09942726,0.365495933,0.258,0.153,1
TPT1,0.099733052,-0.296509217,1,1,1
DUSP6,0.102884869,0.367501998,0.409,0.305,1
RPL11,0.105931981,-0.258862405,1,0.983,1
ZFP36L1,0.108674879,-0.314425028,0.712,0.864,1
ZFP36L2,0.113869252,-0.276521257,0.591,0.729,1
PSAP,0.114531829,-0.407911657,0.227,0.339,1
NFIA,0.114655664,-0.422875886,0.515,0.627,1
ANXA1,0.114874102,0.564826036,0.864,0.763,1
CTSD,0.115440647,-0.456757866,0.576,0.644,1
SFTPC,0.118257772,1.569654999,0.03,0.102,1
MEG3,0.119180574,0.472599458,0.152,0.068,1
MTRNR2L12,0.119885734,-1.271173898,0.97,0.966,1

FAM120AOS,0.120513259,-0.251388282,0.182,0.288,1
SF1,0.122536143,-0.349029638,0.424,0.492,1
DUSP23,0.124818425,-0.276393636,0.621,0.678,1
RGS2,0.128664244,-0.261286744,0.045,0.119,1
AVPI1,0.131849435,-0.268666274,0.182,0.271,1
KLF5,0.136040912,-0.267361258,0.758,0.847,1
CFB,0.136463688,0.269588242,0.212,0.119,1
JUNB,0.137614006,-0.284294563,0.773,0.915,1
POSTN,0.139618979,0.62782915,0.152,0.068,1
RPS3A,0.143570068,-0.298113672,0.955,0.983,1
HIST1H4C,0.148676643,-0.312167913,0.288,0.373,1
TIMP3,0.160006758,0.293443556,0.121,0.051,1
MT-ND3,0.16002043,-0.303687576,1,1,1
RPS27,0.16198872,-0.280290685,1,1,1
DYNLL1,0.162211385,0.253286429,0.5,0.407,1
RPS6,0.164424257,-0.267937309,1,1,1
IFI16,0.167692115,0.333013622,0.47,0.407,1
C12orf57,0.168627586,-0.263931465,0.561,0.593,1
RPS16,0.178681962,-0.279729349,0.985,0.983,1
LINC00472,0.187272636,-0.256078453,0.106,0.186,1
RPL18,0.193796279,-0.263232388,0.97,0.966,1
SLC6A8,0.197173912,0.317416767,0.197,0.119,1
CST3,0.20651366,0.271759089,0.515,0.475,1
LDLR,0.209283698,-0.360296749,0.561,0.627,1
NR4A1,0.213190217,-0.412712644,0.348,0.424,1
MARCKS,0.213699158,0.300354683,0.545,0.407,1
IFRD1,0.21371085,-0.260314904,0.379,0.458,1
AQP3,0.221325175,-0.310031077,0.894,0.881,1
RPL23,0.228431559,-0.258919729,0.894,0.949,1
MT-ND5,0.228871354,-0.327174679,1,0.881,1
DST,0.239540771,0.415306233,0.53,0.475,1
MT-ND1,0.252928307,-0.627377846,1,1,1
RPS4X,0.259842389,-0.32009569,0.985,1,1
ZFP36,0.265963269,-0.325449497,0.803,0.847,1
DNAJA1,0.281588091,0.298883296,0.576,0.508,1
FOSB,0.287240671,-0.478678484,0.848,0.797,1
WWTR1,0.300798563,-0.480573254,0.364,0.373,1
MT-ND2,0.302538254,-0.52184016,0.985,0.949,1
SOX4,0.306778543,-0.275407852,0.742,0.712,1
HIPK3,0.306971174,-0.250287745,0.197,0.254,1
NEAT1,0.337943347,-0.3248245,0.97,0.881,1
MT-ND4,0.340803297,-0.260624197,1,1,1
SLC6A6,0.355413085,0.340749271,0.364,0.305,1
INTS6,0.375087593,-0.400263157,0.273,0.322,1
NDRG1,0.396622528,0.284313325,0.212,0.169,1
TAGLN2,0.444756696,-0.291310704,0.682,0.695,1
ACTB,0.456601228,0.330711401,0.909,0.932,1
CH25H,0.497504564,0.400562692,0.424,0.373,1
CTSS,0.501058133,-0.290484384,0.121,0.153,1
HSPA1B,0.502123608,-0.557679746,0.258,0.288,1

PHLDA2,0.587303201,0.301905135,0.545,0.61,1
SCGB1A1,0.603404309,2.949782886,0.182,0.237,1
MTRNR2L8,0.630369583,-0.657708565,0.712,0.763,1
HSPA1A,0.782831158,-0.712888693,0.424,0.424,1
GDF15,0.802723942,0.710092579,0.303,0.305,1
TIMP1,0.855120756,0.291966897,0.394,0.39,1
